# Green synthesis of silver nanoparticles from *Bacillus subtilis*-mediated feather hydrolysate: antimicrobial, larvicidal against *culex pipiens*, and anticancer activities

**DOI:** 10.1186/s40643-025-00952-y

**Published:** 2025-10-17

**Authors:** Mohammed H. Alruhaili, Samy Selim, Eslam Adly, Mohanned T. Alharbi, Bassam M. Al-ahmadi, Mutasem S. Almehayawi, Soad K. Al Jaouni, Salem S. Salem, Samah H. Abu-Hussien

**Affiliations:** 1 Department of Clinical Microbiology and Immunology, College of Medicine, King Abdulaziz University, 21589 Jeddah, Saudi Arabia; 2 Special Infectious Agents Unit, King Fahad Medical Research Center, King AbdulAziz University, Jeddah, Saudi Arabia; 3 Department of Clinical Laboratory Sciences, College of Applied Medical Sciences, Jouf University, 72388 Sakaka, Saudi Arabia; 4 Department of Zoology, Faculty of Science, Ain Shams University, Cairo, Egypt; 5 Department of Basic Medical Sciences, College of Medicine, University of Jeddah, Jeddah, Kingdom of Saudi Arabia; 6 Department of Biology, Faculty of Science, Taibah University, Madinah, 42353 Kingdom of Saudi Arabia; 7 Department of Emergency Medicine, College of Medicine, King Abdulaziz University, Jeddah, Kingdom of Saudi Arabia; 8 Department of Hematology/Oncology, Chair of Prophetic Medicine Application, king Abdulaziz University Hospital, King Abdulaziz University, 21589 Jeddah, Kingdom of Saudi Arabia; 9 Botany and Microbiology Department, Faculty of Science, Al-Azhar University, Nasr City, Cairo, 11884 Egypt; 10 Department of Agricultural Microbiology, Faculty of Agriculture, Ain Shams University, Cairo, Egypt

**Keywords:** Feather waste bioprocessing, *Bacillus subtilis*, Silver nanoparticles, Antimicrobial activity, Anticancer therapy, Larvicidal activity

## Abstract

This study presents a novel dual-stage bioprocessing approach that transforms poultry feather waste into multifunctional silver nanoparticles (FWH-AgNPs) with enhanced bioactivity. *Bacillus subtilis* degradation of feather waste produced bioactive hydrolysate (FWH) with dramatically altered chemical composition, generating novel compounds including 9,12,15-octadecatrienoic acid methyl ester (25.66%) and cyclopropaneoctanoic acid methyl ester (23.02%). The FWH effectively synthesized spherical AgNPs (30–69 nm) with strong colloidal stability (−44.5 mV zeta potential) and characteristic surface plasmon resonance (420 nm). FWH-AgNPs demonstrated superior antimicrobial efficacy with 4–eightfold improved minimum inhibitory concentrations against *Pseudomonas aeruginosa* (125 μg/mL), methicillin-resistant *Staphylococcus aureus* (250 μg/mL), *Aspergillus brasiliensis* (275 μg/mL), and *Candida albicans* (125 μg/mL). Comparable enhancements were also observed for *Serratia marcescens* (300 μg/mL) and *Bacillus cereus* (325 μg/mL), further confirming the broad-spectrum antimicrobial potential of FWH-AgNPs. Anticancer evaluation revealed selective cytotoxicity toward MCF-7 breast cancer cells (IC₅₀: 294.7 μg/L) with favorable selectivity index (2.68) over normal fibroblasts. Optimized FWH-AgNPs achieved 87.38% larvicidal mortality against *Culex pipiens*, validated through Box-Behnken methodology. Mechanistic studies revealed systematic disruption of larval metabolism, including protein depletion, carbohydrate exhaustion, and acetylcholinesterase inhibition, coupled with severe midgut epithelial damage. Molecular docking identified α1-sitosterol as the primary bioactive compound with strong binding affinities to antimicrobial targets (−7.1 to −7.4 kcal/mol) and cancer receptors (−7.0 to −9.5 kcal/mol). This integrated approach successfully addresses environmental waste management while generating high-value nanomaterials for biomedical and vector control applications, establishing a new paradigm for circular bioeconomy applications.

**Graphical abstract:**

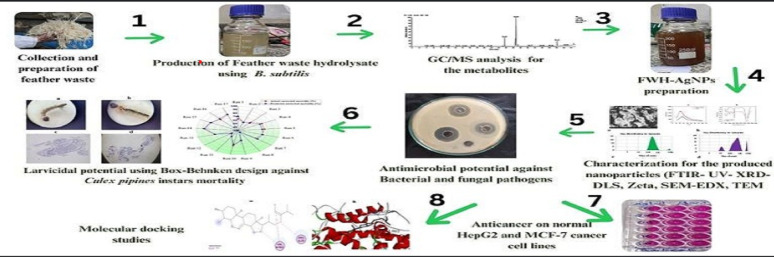

**Supplementary Information:**

The online version contains supplementary material available at 10.1186/s40643-025-00952-y.

## Introduction

The global poultry industry generates enormous quantities of feather waste, with annual production exceeding 8.5 million tons worldwide (Karuppannan et al. 2021). This by-product constitutes approximately 5–7% of the total chicken weight and presents significant disposal challenges due to its recalcitrant structure, primarily composed of α1-keratin (90–95%), which resists conventional degradation processes (Banasaz and Ferraro 2024). Traditional management approaches including landfilling, incineration, and conversion to low-value feedstock are increasingly problematic due to environmental concerns, regulatory limitations, and inefficient resource utilization (Durak 2023). Consequently, there is growing interest in developing sustainable valorization strategies that transform this abundant waste stream into value-added products.

Microbial biotransformation offers a promising approach for feather waste utilization through enzymatic hydrolysis. Among various keratin-degrading microorganisms, *B. subtilis* has emerged as particularly effective due to its robust keratinolytic enzyme production system, comprising keratinases, proteases, and disulfide reductases (Das et al. 2024). Recent studies have demonstrated that microbial hydrolysis not only facilitates waste degradation but can also enhance the bioactive potential of the resulting hydrolysate by liberating or generating compounds with antimicrobial, antioxidant, and anticancer properties (Raj et al. 2022).

In parallel, green nanotechnology has gained significant attention as an environmentally responsible alternative to conventional nanoparticle synthesis (Salem 2023; Salem and Fouda 2021). Traditional methods often involve toxic chemicals and harsh conditions, raising biocompatibility and environmental concerns (Ahmed et al. 2022). Green synthesis utilizes biological extracts rich in reducing and stabilizing biomolecules, enabling nanoparticle formation under mild and sustainable conditions (Salem et al. 2022; Soliman et al. 2024; Kirubakaran et al. 2025; Salem 2022). Among nanomaterials, silver nanoparticles (AgNPs) are widely studied due to their broad-spectrum antimicrobial efficacy, anti-inflammatory activity, and emerging roles in vector control and cancer therapy (Al-Rajhi et al. 2022; Bamal et al. 2021; Said et al. 2024).

The integration of waste valorization and green nanotechnology presents a compelling dual-benefit strategy addressing both environmental waste burdens and the need for biocompatible nanomaterials (Omran and Baek 2022; Zhu et al. 2020). While plant-based wastes such as fruit peels, crop residues, and food by-products have been widely employed in the green synthesis of AgNPs due to their phytochemical richness, they often lack the unique amino acid and peptide profile provided by keratinous materials. Unlike plant matrices, feather waste rich in structural proteins offers a distinct bio-reduction environment, particularly when pretreated via microbial hydrolysis. However, the utilization of feather waste for AgNPs synthesis remains sparse in the literature, with few studies addressing the role of microbial transformation in enhancing nanoparticle functionality. This study fills this gap by demonstrating how *B. subtilis*-mediated hydrolysate not only acts as a reducing and stabilizing agent but also contributes to improved biological efficacy of the resulting AgNPs., the potential of microbially hydrolyzed feather waste as a novel, protein-rich precursor for AgNPs synthesis remains underexplored. To date, no comprehensive study has demonstrated how microbial processing of feather waste can modulate the precursor’s biochemical profile and impact the synthesis quality and multifunctional performance of the resulting nanoparticles.

This study addresses this gap by investigating the microbial transformation of poultry feather waste using *B. subtilis*, followed by the green synthesis of silver nanoparticles from the resulting feather waste hydrolysate (FWH-AgNPs). We hypothesized that microbial processing would enhance the hydrolysate’s bioactive profile, thereby influencing nanoparticle synthesis efficiency and functional activity. Our specific objectives were to characterize the biochemical changes in feather waste after microbial degradation using GC–MS; synthesize and characterize FWH-AgNPs using spectroscopic, microscopic, and physicochemical tools; optimize synthesis parameters via Box–Behnken design; evaluate the antimicrobial, larvicidal, and anticancer potential of the synthesized AgNPs followed by exploring the molecular interactions through in silico docking studies.

By demonstrating the conversion of feather waste into multifunctional nanomaterials with applications in healthcare, agriculture, and vector control, this study not only contributes to the circular bioeconomy but also provides a novel strategy for enhancing the utility and performance of green-synthesized nanomaterials. Importantly, it fills a critical gap in existing research by presenting feather waste hydrolysate as a viable, underutilized platform for developing high-impact nanotechnological applications.

## Materials and methods

### Collection of feather waste

A total of 25 poultry waste samples—consisting of feathers, meat, and bones—were aseptically collected from slaughterhouses in Cairo, Egypt, using sterile plastic bags. The samples were transported on ice to the Biology Laboratory at the Faculty of Agriculture, Ain Shams University, and subsequently stored at 4 °C for further investigation.

### Microorganisms and media used

The *B. subtilis* strain was acquired from an earlier research project and maintained on nutrient medium (Mansour et al. 2023). The antimicrobial properties of the feather waste silver nanoparticles (FWH-AgNPs) were tested against four bacterial strains (*Pseudomonas aeruginosa* ATCC-27853, Methicillin-Resistant *Staphylococcus aureus, Serratia marcescens* ATCC 14756, *Bacillus cereus* ATCC 11778) and two fungal strains (*Aspergillus brasiliensis* ATCC-16404 and *Candida albicans* ATCC-10231). Nawah Scientific, located in Cairo, Egypt (https://nawah-scientific.com), supplied these test microorganisms. The tryptone-glucose yeast (TGY) medium was used to cultivate the bacterial cultures, while The potato-dextrose agar (PDA) medium was used to cultivate the fungal cultures. Every culture was stored at 4°C until additional testing was required.

### Standard inoculum

Using a rotary shaker (Lab-line Ltd.), the bacteria were cultured in nutritional medium at 30°C for 24 h while being shaken at 120 rpm to create a typical inoculum of *B. subtilis*. This produced a suspension of around 7.0 × 10^5^ viable cells/mL for use in investigations (Abu-Hussien and Mohamed 2020).

### Feather waste hydrolysate production using *B. subtilis*

FWH was produced according to a previous study (Saeed et al. 2025). Briefly, chicken feathers were thoroughly washed, boiled for 20 min, pressed to remove moisture, dried at 90°C for 22 h., and ground into a fine powder. For the hydrolysis medium, 100 mL of TGY medium containing 15 g/L feather powder was placed in 250 mL Erlenmeyer flasks. The pH was adjusted to 8.0, and the medium was autoclaved at 121°C for 20 min. After cooling, flasks were inoculated with 5% (v/v) *B. subtilis* standard inoculum and incubated at 37°C for 24 h. with shaking at 200 rpm. Following incubation, cultures were centrifuged at 10,000 rpm for 15 min at 4°C, and the cell-free supernatant was collected as the feather waste hydrolysate (Nassar et al. 2015).

### Gas chromatography GC/MS analysis

The FWH samples were first concentrated through evaporation, then re-dissolved in methanol in preparation for GC–MS analysis. This procedure was conducted to detect intermediate metabolites resulting from the biodegradation of feather waste by *B. subtilis*. The chemical composition was analyzed using a Thermo Scientific Trace GC-TSQ mass spectrometer (Austin, TX, USA) fitted with a TG–5MS capillary column (30 m × 0.25 mm, 0.25 µm film thickness). The temperature program began at 50°C, increased gradually at 5°C per minute to reach 250°C (held for 2 min), then rapidly rose to 300°C at a rate of 30°C per minute and was held for another 2 min. The injector and MS transfer line were set to 270°C and 260°C, respectively. Helium was used as the carrier gas at a constant flow of 1 mL/min. A 4-min solvent delay was applied before automatically injecting 1 µL of the diluted sample in split mode using the Autosampler AS1300. Mass spectral data were collected using an electron ionization source at 70 eV, with a scan range from m/z 50 to 650 in full scan mode, and the ion source temperature maintained at 200°C. Identification of metabolites was based on spectral comparisons with entries in the WILEY 09 and NIST 14 libraries (Abd-Elhalim et al. 2023; El-Tabakh et al. 2025).

### Synthesis of silver nanoparticles using FWH

**Fig. 1:**
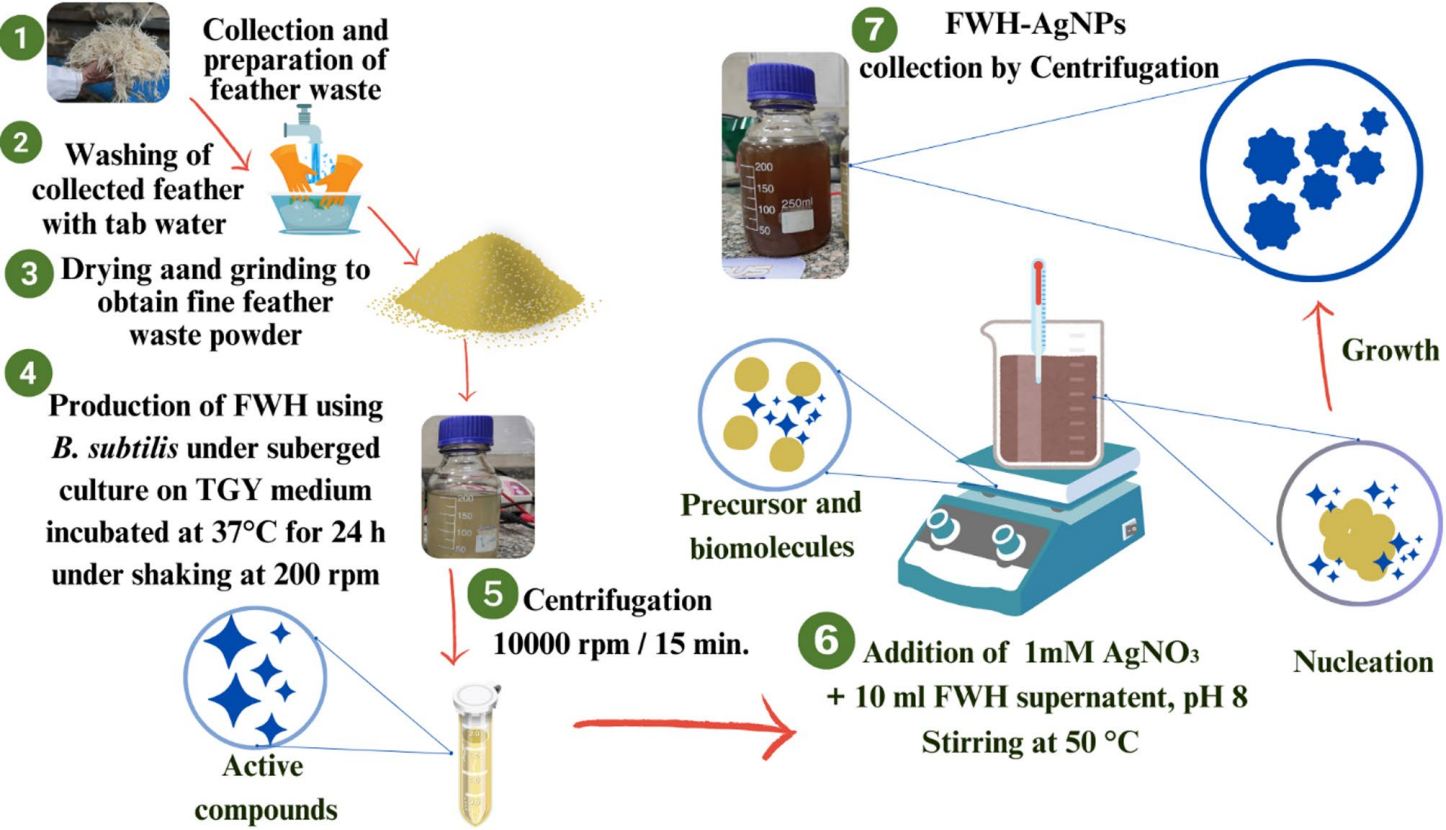
Biosynthesis of silver nanoparticles from feather waste using *B. subtilis*

To synthesize silver nanoparticles, a 50 mL aliquot of 1 mM AgNO_3_ solution was added dropwise to 2 mL of feather waste hydrolysate (FWH) previously prepared via enzymatic degradation using *B.subtilis*. The reaction was carried out in a 100 mL glass beaker placed in a rotary shaker incubator (Lab-line Ltd., USA) set at 200 rpm and 30 °C. The mixture was incubated overnight to prevent photoreduction. A control sample containing only the hydrolysate without AgNO₃ was formed under identical conditions. The formation of feather waste hydrolysate-mediated silver nanoparticles (FWH-AgNPs) was visually indicated by a color change from pale-yellow to light-brown, consistent with AgNPs synthesis (Amr et al. 2023). The resulting nanoparticles were collected by centrifugation and stored for further characterization and biological evaluations (Fig. 1).

### Characterization of FWH-AgNPs

The FWH-AgNPs underwent comprehensive characterization using multiple analytical techniques. Optical properties of the nanoparticles were examined using UV–Visible spectroscopy with a UV Analyst-CT 8200 spectrophotometer, scanning wavelengths from 200 to 800 nm. To identify functional groups, Fourier Transform-Infrared Spectroscopy was employed using a Shimadzu Tracer-100 system, with measurements taken across 1000–4000 nm using 4 cm⁻^1^ resolution and a 2.4 refractive index. Particle size distribution was determined through Dynamic Light Scattering analysis using a Zetasizer Nano ZS (Nano Series HT) manufactured by Malvern Instruments, UK. The surface charge characteristics were assessed by measuring zeta potential with a NICOMPTM-380 ZLS-Analyzer, which utilizes phase analysis light-scattering at 286 kHz frequency and 1.54 nm wavelength, with all measurements conducted at 30°C. For morphological examination, Field Emission Scanning-Electron Microscopy was performed using a JEOL-JSM-7800F operated at 15 kV. The elemental composition was determined through Energy Dispersive X-ray Spectroscopy (HR-SEM–EDX) using a VEGA 3-T Scan operated at 20 kV. Crystalline structure analysis was performed via X-ray Diffraction using a D2-Phaser Bruker 2nd-Generation diffractometer. The samples were analyzed on glass substrates across various Bragg angles using Cu Kα radiation (λ = 1.5406 Å). The full width at half maximum values were extracted from the diffraction patterns and converted to radians. The crystallite size was subsequently calculated using the Scherrer equation, incorporating a shape factor (K = 0.9) and the Bragg angle expressed in radians. High-resolution Transmission Electron Microscopy (HR-TEM, JEOL JEM-2100, 200 kV, 2838 cps) was employed to obtain detailed images of the nanoparticle structure. This analysis provided valuable insights into the nanoscale structural properties of the materials, revealing variations based on peak broadening phenomena.

### Antimicrobial properties of FWH-AgNPs

#### Antimicrobial test using the agar well-diffusion technique

The antimicrobial activity of feather waste hydrolysate-silver nanoparticles (FWH-AgNPs) was evaluated against *P. aeruginosa* (ATCC 27853), methicillin-resistant *S. aureus* (MRSA), *C. albicans* (ATCC 10231), and *A. brasiliensis* (ATCC 16404) using the agar well diffusion method. Mueller–Hinton agar (MHA) was used for bacterial strains, and Potato dextrose agar (PDA) for fungi. Plates were inoculated with 50 µL of microbial suspension standardized to 10⁶ CFU/mL. Punched wells of 6 mm in diameter were filled with 100 µL of FWH-AgNPs at doses between 400—25 µg/mL for bacteria and 1000—25 µg/mL for fungus. Gentamycin (30 µg/disc) and fluconazole (25 µg/disc) served as positive controls for bacterial and fungal pathogens, respectively, while distilled water was used as a negative control. The plates were incubated at 37 °C for 24 h for bacterial strains and at 25 °C for 72 h for fungal strains. Inhibition-zone diameters (IZDs) were measured in millimeters (mm) using a digital-caliper, and experiments were performed in triplicate (Abd-Elhalim et al. 2023).

#### Minimum inhibitory-concentrations (MIC), minimum bactericidal-concentrations (MBC) and minimum fungicidal-concentrations (MFC)

The MIC, MBC, and MFC were determined utilizing the broth microــdilution method in 96-well microplates. Serial two-fold dilutions of FWH-AgNPs and unmodified feather waste hydrolysate (FWH) were prepared in tryptone-glucose-yeast extract (TGY) broth. A microbial inoculum equivalent to 0.5 McFarland standard was added to each well. After incubation at 37 °C for 24 h (bacteria) or 25 °C for 48–72 h (fungi), MIC was defined as the lowest dose with no visible turbidity. For MBC and MFC determination, 10 µL from wells showing no visible-growth were subcultured onto MHA (bacteria) or PDA (fungi) and incubated again. MBC/MFC was defined as the lowest concentration that resulted in ≥ 99.9% killing of the initial inoculum. All assays were conducted in triplicate (Farouk et al. 2023).

#### Larvicidal potential against culex pipines larvae

##### Optimization of FWH-AgNPs larvicidal activity by box behnken design

To optimize the larvicidal efficacy of feather waste hydrolysate-derived silver nanoparticles (FWH-AgNPs), a Box–Behnken design (BBD) (Saeed et al. 2025) was employed using three independent variables: FWH-AgNP concentration (A: 250, 500, and 1000 µg/L corresponding to coded levels -1, 0, and + 1, respectively), exposure time (B: 24, 48, and 72 h; coded as -1, 0, + 1), and pH (C: 6, 7, and 8; coded as -1, 0, + 1). This three-level, three-factor design generated a total of 17 experimental runs, including five replicates at the center point to assess experimental error and reproducibility (Table 1). Each experimental run involved exposing second and third instar *C. pipiens* larvae to a specific combination of the three variables under controlled laboratory conditions. For each treatment, five replicates were conducted, and larval mortality was recorded at the end of the specified exposure period. Corrected mortality rates were calculated using Abbott’s formula to account for natural mortality in the control group as follows (Abbott 1925).

1 $$\text{Corrected Mortality }\left(\text{\%}\right)=\frac{T-C}{100-C} X 100$$

where T = percent mortality in the treatment and C = percent mortality in the control. The actual and predicted values for mortality and corrected mortality were used to fit a quadratic response surface model to determine the most significant factors and interactions influencing larvicidal activity.

**Table 1:** Box–Behnken experimental design matrix for FWH-AgNPs against *C. pipiens*

Run	FWH-AgNPs Conc (A) (µg/l)	Exposure time (B) (h)	pH (C)
1	250 (−1)	48 (0)	6 (−1)
2	250 (−1)	72 (+ 1)	6 (−1)
3	500 (−1)	48 (0)	6 (−1)
4	250 (−1)	48 (0)	6 (−1)
5	250 (−1)	48 (0)	6 (−1)
6	500 (−1)	24 (−1)	6 (−1)
7	250 (−1)	48 (0)	6 (−1)
8	1000 (+ 1)	48 (0)	8 (+ 1)
9	500 (0)	72 (+ 1)	7 (0)
10	500 (0)	48 (0)	7 (0)
11	250 (−1)	48 (0)	6 (−1)
12	500 (0)	24 (−1)	7 (0)
13	1000 (+ 1)	72 (+ 1)	8 (+ 1)
14	1000 (+ 1)	24 (−1)	8 (+ 1)
15	500 (0)	24 (−1)	7 (0)
16	1000 (+ 1)	48 (0)	8 (+ 1)
17	1000 (−1)	72 (+ 1)	6 (−1)

Concentration–response relationships were analyzed using probit regression analysis following established protocols (Finney 1971). Mortality data were transformed using the probit function Φ⁻^1^(P), where P represents the mortality proportion and Φ⁻^1^ is the inverse standard normal cumulative distribution function. The linear relationship between log₁₀ concentration and probit-transformed mortality (Russell and Robertson 1979) was modeled as:

2 $$ P = \alpha + \beta .\log_{10} \left( {concentration} \right) $$

where α and β are regression parameters estimated by weighted least squares. Median lethal concentration (LC₅₀) and 90% lethal concentration (LC₉₀) (Mansour et al. 2023) values were calculated as:

3 $$ {\text{IC}}_{50} { } = { }10^{{\frac{{ - {\alpha } + { }5}}{{\upbeta }}}} $$

4 $$ {\text{LC}}_{90} { } = { }10^{{\frac{{ - {\alpha } + { }6.82}}{{\upbeta }}}} $$

Model adequacy was assessed using Pearson's chi-square goodness-of-fit test (χ^2^), coefficient of determination (R^2^), and residual analysis. Acceptance criteria included R^2^ ≥ 0.85, χ^2^ p-value > 0.05, and randomly distributed residuals. Confidence intervals (95%) for LC values were calculated using the delta method. Zero and 100% mortality values were adjusted using Abbott's correction where necessary.Results are reported as mean ± standard error with significance set at p < 0.05. The design matrix did not include control treatments, as controls are not part of the polynomial response surface model. For bioassay quality control conducted outside the BBD, a concurrent negative control (untreated *C. pipiens* larvae in dechlorinated water) was included in each run to estimate natural mortality; mortality data were corrected using Abbott’s formula when needed. Assays were repeated if control mortality exceeded 10%. No positive control was incorporated into the RSM matrix.

### Biochemical activities of *C. pipines* larvae

Twenty-five second- and third- instar *C. pipiens* larvae were put in 100 mL beakers with different doses of FWH and FWH-AgNPs for the purpose of conducting larval bioassays. Breadcrumbs were given to the larvae, which were kept at 25 to 30 °C with a 14:10 light–dark cycle for 24 h. The number of hours between treatment and larval death was noted. Dead larvae were counted following each experiment, and the negative control was distilled water. Every test was run in triplicate, including the controls. Using Abbott's methodology, mortality percentages were computed and modified. Experimental containers were kept at ambient temperature and left undisturbed during the test period. (% test mortality − % control mortality) / (100 − % control mortality) × 100 (Ga'al et al. 2018; Abu-Hussien et al. 2024).

#### Morphological and histopathological analysis of *C. pipiens*

FWH and FWH-AgNPs were made according to the instructions. Samples were kept at 25 °C for 72 h in 200 mL glass beakers with cotton mesh coverings, each holding 25 mosquito larvae. Every therapy was carried out in triplicate. Using a Labomed microscope at 40X and 100X magnifications, morphological alterations were investigated. Slides containing dead larvae were used for close inspection. The larvae, both control and treated, were preserved in 3–5% formalin, dried with ethyl alcohol, cleaned with xylene, and then embedded in paraplast for histological purposes. Using a Labomed microscope, the mid-gut areas were inspected and photographed after Sects. (7 µm thick) were stained with haematoxylin and eosin (Mansour et al. 2023).

## Biochemical disruption of *C. pipines* larvae

### Total protein content

The Pierce™ BCA Protein Assay Kit (Thermo Sci., Product No. 23225 or 23,227) was used to measure the total protein content. Fifty (50) μL of the test sample or protein standard (including blank) was added to microcentrifuge tubes for each experiment. Next, 450 μL of distilled water, 100 μL of 0.15% sodium deoxycholate, and 100 μL of 72% trichloroacetic acid were added. Samples were incubated at ambient temperature for 10 min before being centrifuged for 15 min at 10,000 rpm. The particle was again suspended in 50 μL of 5% SDS in 0.1 N NaOH, and the supernatant was disposed of. Following the addition of 1 mL of BCA reagent, the tubes were incubated for 30 min at 37°C. Protein content was represented as the quantity per 25 larvae, and absorbance was calculated at 562 nm (Mansour et al. 2023).

#### Determination of total carbohydrate content

A standard curve was created by serially diluting a glucose stock solution (1 mg/mL) from 12.5 to 400 µg/mL. In order to collect the supernatant and dilute it 1:1 with distilled water, the larvae were homogenised in a sterile mortar and centrifuged for 15 min at 10,000 rpm. For analysis, 100 µL of 75% sulphuric acid was combined with 50 µL of each diluted sample, and then 200 µL of anthrone reagent (5 mg in 100 µL ethanol + 2.4 mL of 75% sulphuric acid) was added. After 5 min of heating at 100°C, the mixture was allowed to cool at ambient temperature for an additional 5 min. A microplate reader was then used to detect absorbance at 578 nm after 100 µL of each sample had been put to a 96-well plate. Three separate experiments were used for the measurements, and the findings were reported as means ± standard deviation.The reference for calculating the quantity of soluble carbohydrates was glucose (Mansour et al. 2023).

#### Acetylcholinesterase (AChE) activity assay

An inhibitory experiment was used to evaluate the activity of acetylcholinesterase (AChE), with donepezil (5 mM) serving as the positive control. 10 µL of indicator solution (0.4 mM in 100 mM Tris buffer, pH 7.5) and 20 µL of AChE enzyme (0.02 U/mL in 50 mM Tris buffer, pH 7.5, including 0.1% bovine serum albumin) were added to each well of a 96-well plate. Next, 140 µL of buffer and 20 µL of the test sample or standard solution were added. For fifteen minutes, the plate was incubated at room temperature. Following the addition of 10 µL of cholinergic iodide (0.4 mM) to each well, the plate was left to incubate for 20 min at room temperature in the dark. A microplate reader was used to detect absorbance at 412 nm. Means ± standard deviation were used to express the findings (Mansour et al. 2023).

#### Invitro anticancer activity of FWH on skin normal cells (HSF) and MCF-7 cell line

We purchased HSF and MCF-7 breast cancer cell lines from Nawah Scientific Inc. in Mokatam, Cairo, Egypt. In order to maintain ideal growth conditions, the cells were cultivated in Dulbecco's Modified-Eagle Medium (DMEM) supplied with 10% heat-inactivated foetal bovine serum and antibiotics (100 mg/mL streptomycin and 100 u/mL penicillin). The cultures were maintained at 37°C in a humidified environment with 5% CO2. The MTT test technique was used with quantities of FWH-AgNPs (1000, 500, 250, 125, 62.5, and 31.25 µg/mL) to assess cell viability (Khaled et al. 2025).

## Molecular docking studies

### Ligand preparation

The molecular structures of selected bioactive compounds including fatty acid methyl esters and α1-sitosterol—were retrieved from the Pub-Chem database in SDF-format. Ligands were energy-minimized using Avogadro 1.2.0 software with the MMFF94 force field to achieve optimal conformations for docking (Hanwell et al. 2012).

### Protein target selection and preparation

Six protein targets were selected to assess antimicrobial and anticancer activity, FtsZ (*S. aureus*, UniProt ID: P0A031), CzcS (*P. aeruginosa*, UniProt ID: A0A0N9ZXD2), WalK (*S. aureus*, UniProt ID: Q9RDT3), Androgen receptor (AR) (*Homo sapiens*, UniProt ID: P10275) and Estrogen receptor α1 (ESR2) (*Homo sapiens*, UniProt ID: Q92731). The protein structures were downloaded from the RCSB Protein Data Bank or modeled if not available. Using AutoDock Tools 1.5.7, all proteins were prepared by deleting heteroatoms and water molecules, adding polar hydrogens, and allocating Gasteiger charges. Grid boxes were generated around the known or predicted active sites (Morris et al. 2009).

### Docking procedure

AutoDock Vina was used to dock, and the exhaustiveness level was set at 8. For each ligand–protein pair, the best-ranked binding pose based on binding free energy (ΔG, kcal/mol) was selected for further analysis. The docking protocol was repeated for reproducibility and to ensure consistent scoring (Eberhardt et al. 2021).

### Visualization and analysis

The BIOVIA Discovery Studio Visualiser 2020 was used to visualise protein–ligand interactions (https://discover.3ds.com/discovery-studio-visualizer-download). Important interactions were found and examined, including π-π stacking, hydrophobic contacts, and hydrogen bonds. Binding affinities (ΔG values) were recorded and compared across all compounds and targets.

### Chemicals

Sigma-Aldrich (St. Louis, MO, USA) supplied all analytical-grade chemicals and reagents utilised in the investigation.

### Statistical analysis

The data are shown as mean ± standard deviation (SD), and all tests were carried out in triplicate (n = 3). Using OriginPro and Design Expert 12 software, a two-way ANOVA with a significance level of P < 0.05 was used for statistical analysis. Tukey's post hoc test was used for multiple comparisons at the same level of significance (P < 0.05) (Keselman and Rogan 1977).

## Results and discussion

### GC–MS analysis of bioactive compounds following *B. subtilis* degradation of feather waste

**Fig. 2:**
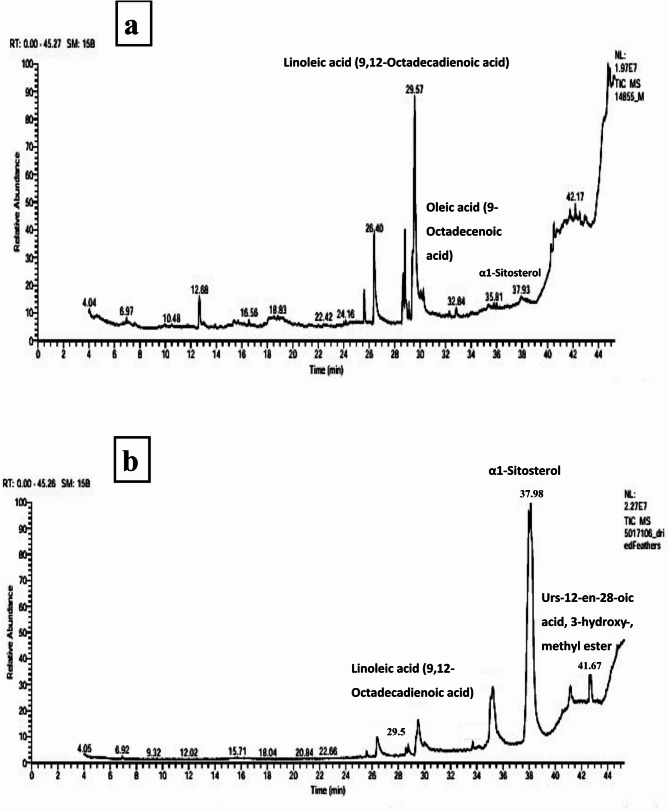
Major bioactive compounds identified by GC–MS in feather waste **a** before and **b** after microbial degradation by *B. subtilis*

GC–MS analysis of feather waste before and after bacterial degradation by *B. subtilis* revealed significant shifts in the composition of bioactive compounds (Table 2** and **Fig. 2). The most abundant compound in the untreated sample was α1-sitosterol (33.21%), which decreased to 21.53% following degradation, yet remained a major constituent. Several bioactive metabolites not present in the original sample were newly detected after treatment, including 9,12,15-octadecatrienoic acid methyl ester (25.66%), urs-12-en-28-oic acid, 3-hydroxy-, methyl ester (8.49%), and cyclopropaneoctanoic acid methyl ester (23.02%). These compounds are known or suggested to possess antioxidant, anticancer, or antimicrobial activities as reported by Annamalai and Kasilingam (2024) (Annamalai and Kasilingam H: BIO-PROSPECTING 2024) who identified similar cyclopropane fatty acid derivatives as potent antimicrobial agents in microbially-transformed agricultural waste. In contrast, linoleic acid declined from 4.20% to 2.29%, and oleic acid (3.85% in the untreated sample) was undetected. Overall, the relative area percentage of other minor or unidentified compounds decreased from 58.74% in the untreated sample to 19.01% after degradation. This transformation aligns with previous studies by Sharma and Gupta (2024)(Sharma and Gupta 2024) who stated that *B. subtilis* selectively transformed feather waste components into novel or enriched metabolites with potential pharmaceutical applications. *B. subtilis* functions through a three-phase mechanism: (1) enzymatic keratin degradation via keratinases and proteases that liberate amino acids (particularly cysteine, methionine, and tyrosine) serving as reducing agents for Ag⁺ → Ag⁰ conversion, (2) metabolite biotransformation that converts substrates into secondary metabolites with enhanced electron-donating capabilities, and (3) silver ion reduction and nucleation where thiol-containing compounds and fatty acid metabolites reduce Ag⁺ ions while acting as capping agents to control particle size. This biocatalytic approach offers sustainable reducing agents, enhanced bioactivity through bacterial metabolites, waste valorization of agricultural byproducts, and environmentally benign operation, demonstrating how microbial metabolism can be harnessed for sustainable nanotechnology applications while transforming waste into high-value nanomaterials (Caulier et al. 2019).

**Table 2:** Major bioactive compounds identified by GC–MS in feather waste before and after microbial degradation by *B. subtilis*

Compound name	Retention time (min) before	Area % before	Retention time (min) after	Area % after	Molecular formula	Molecular weight
α1-Sitosterol	37.98	33.21	37.98	21.53	C_₂₉_H_₅₀_O	414.7
9,12,15-Octadecatrienoic acid, methyl ester	ND	ND	28.72	25.66	C_19_H_₃₂_O₂	292.5
Cyclopropaneoctanoic acid, methyl ester	ND	ND	28.79	23.02	C_11_H_20_O_2_	184.3
Linoleic acid (9,12-Octadecadienoic acid)	29.51	4.20	29.51	2.29	C_18_H_32_O_2_	280.4
Oleic acid (9-Octadecenoic acid)	29.78	3.85	ND	ND	C_18_H_32_O_2_	282.5
Urs-12-en-28-oic acid, 3-hydroxy-, methyl ester	ND	ND	41.67	8.49	C_18_H_32_O_3_	474.7

### Visual and spectral characterization of FWH-AgNPs synthesized from feather waste hydrolysate by *B. subtilis*

**Fig. 3:**
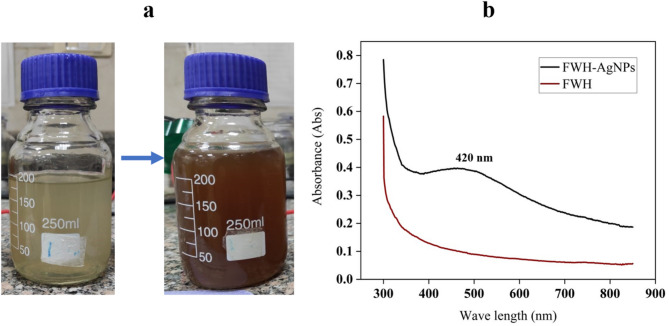
**a** Visual confirmation of Ag-NPs synthesis indicated by a color change in *B. subtilis*-derived FWH, transitioning from yellowish (before AgNO₃ addition) to dark brown (after reduction). **b** UV–Vis absorption spectra of FWH and FWH-AgNPs

The biosynthesis of AgNPs using *B. subtilis*-mediated FWH was visually confirmed by a distinct color change (Fig. 3). Initially yellowish due to bacterial metabolites, the FWH solution turned dark brown upon the addition of silver nitrate (AgNO₃), indicating the reduction of Ag⁺ ions and formation of silver nanoparticles. This color change is attributed to surface plasmon resonance (SPR), a hallmark of AgNPs. Further confirmation was obtained through UV–Vis spectroscopy (Fig. 3). Measurements were conducted against distilled water (blank) with baseline correction across the 200–800 nm range. The FWH-AgNPs exhibited a strong SPR absorption peak centered at 420 nm (λmax = 420 nm) with a maximum absorbance of 0.85, aligns with the findings of Mukhtar et al. (2018) (Mukhtar et al. 2018) who reported similar optical properties for biogenically synthesized AgNPs. with the optical profile of colloidal AgNPs. The full width at half maximum was approximately 80 nm, suggesting moderate size distribution with relatively monodisperse nanoparticles. In contrast, untreated FWH showed absorption peaks at 280 nm (aromatic amino acids) and 260 nm (nucleic acids), with no SPR features in the 400–500 nm range. Based on the extinction coefficient for spherical AgNPs at 420 nm (ε ≈ 1.5 × 10⁹ M⁻^1^ cm⁻^1^), the estimated particle concentration was approximately 3.5 × 10⁻^11^ M.

### Structural and functional group characterization of FWH-AgNPs synthesized from hydrolyzed feather waste by *B. subtilis*

**Fig. 4:**
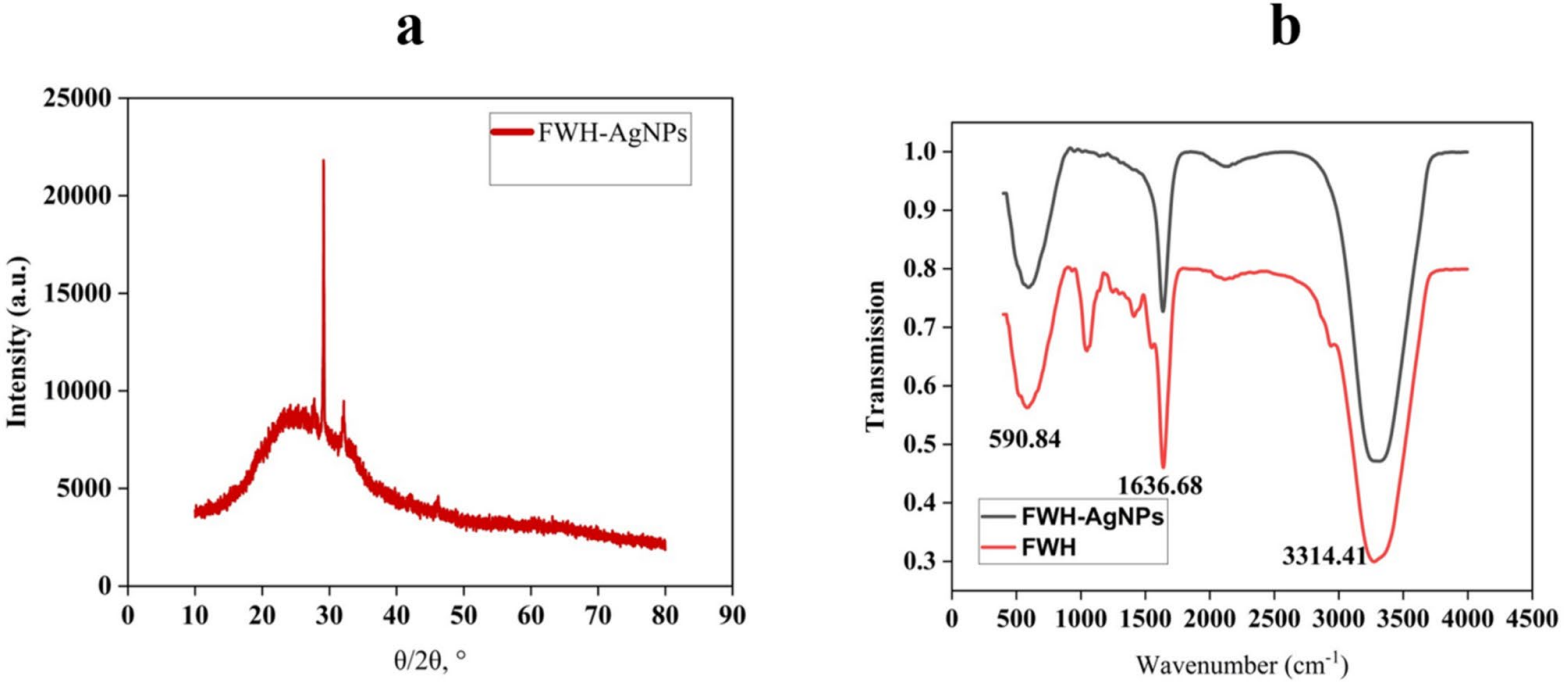
**a** X-ray diffraction (XRD) pattern of FWH-AgNPs. **b** FTIR of FWH and FWH-AgNPs

The structural and functional characteristics of silver nanoparticles synthesized using *B. subtilis*-mediated FWH were investigated through X-ray diffraction (XRD) **supplementary data (S1)** and Fourier-transform infrared (FTIR) spectroscopy (Fig. 4). The XRD profile of FWH-AgNPs revealed distinct diffraction peaks at approximately 2θ = 38.1°, 44.3°, 64.4°, and 77.5°, corresponding to the (111), (200), (220), and (311) planes of face-centered cubic (fcc) silver. The sharpness and intensity of the diffraction peaks indicate high crystallinity of the nanoparticles. The average crystallite size of the FWH-AgNPs was calculated using the Debye–Scherrer equation.

5 $$D=K\lambda /\beta cos \theta $$

where *D* is the crystallite size, *K* is the shape factor (0.9), *λ* is the X-ray wavelength (0.15406 nm), *β* is the full width at half maximum (FWHM) of the diffraction peak in radians, and *θ* is the Bragg angle. Based on the (111) peak, the mean crystallite size was estimated to be 43.06 nm, consistent with the nanoscale features observed by TEM. These results confirm the successful formation of highly crystalline silver nanoparticle as indexed in the JCPDS card No. 04–0783 and comparable to those reported by Al-Mahmud et al. (2024) (Al-Mahmud et al. 2024).These well-defined reflections confirm the formation of crystalline silver nanoparticles and underscore the dual role of FWH as both reducing and stabilizing agent. FTIR spectral analysis further elucidated the functional groups involved in the synthesis process. A broad O–H stretching band (3300–3500 cm⁻^1^) observed in the FWH spectrum decreased in intensity in the FWH-AgNPs, indicating participation of hydroxyl groups in Ag⁺ ion reduction consistent with mechanisms proposed by Lateef et al. (2024) (Lateef et al. 2024). Aliphatic C–H stretching bands near 2900 cm⁻^1^ showed negligible variation, suggesting limited interaction in this region. A slight shift in the carbonyl (C = O) stretching band from 1650 cm⁻^1^ in FWH to 1642 cm⁻^1^ in FWH-AgNPs implies potential coordination between silver and carbonyl-containing moieties. Additional changes observed between 1000 and 1500 cm⁻^1^, corresponding to C–O (alcohols, esters) and C–N (amines or amides) vibrations, suggest their involvement in nanoparticle stabilization. Collectively, these findings indicate that hydroxyl, carbonyl, and nitrogen-containing functional groups in the FWH matrix were instrumental in both the bioreduction of silver ions and the stabilization of the synthesized nanoparticles(Sharma et al. 2023).

### SEM–EDX analysis

**Fig. 5:**
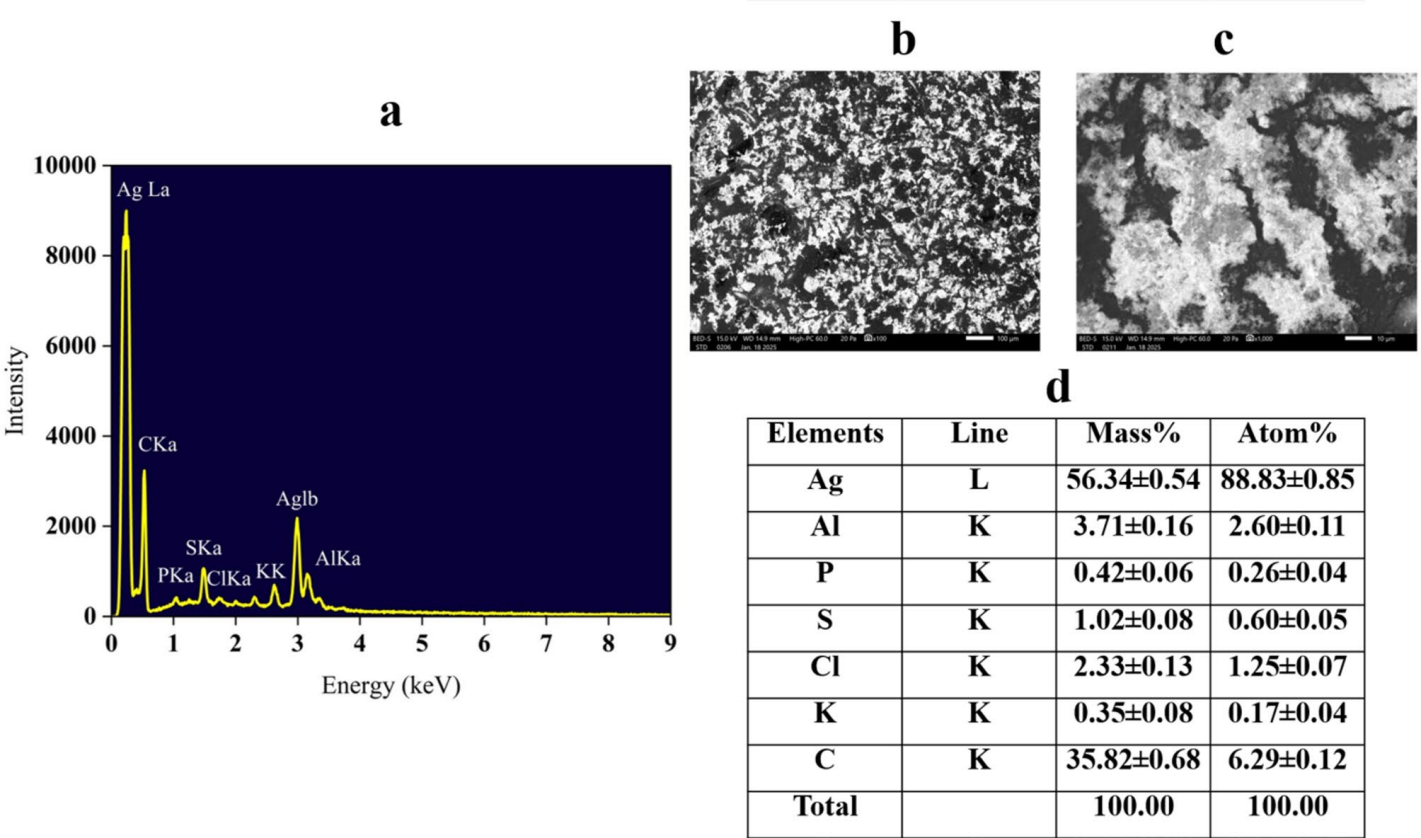
**a**, **d** EDX spectra of FWH-AgNPs. **b**, **c** SEM images of FWH-AgNPs at 2000 × magnification (scale bar: 100 nm)

Scanning electron microscopy (SEM) images (Fig. 5) revealed agglomerated nanoparticle clusters with irregular shapes, consistent with the formation of AgNPs. These aggregates were observed at 2000X magnification, with a reported scale bar of 100 nm, assumed accurate per instrument settings; however, independent calibration is recommended to confirm spatial resolution. Energy-dispersive X-ray spectroscopy (EDX) spectra (Fig. 4a, d) confirmed elemental silver as the predominant component, comprising 56.34 ± 0.54 wt% (88.83 ± 0.85 atom%). This value, while supportive of AgNP synthesis, is slightly lower than typically reported for highly purified AgNPs, likely due to residual organic and inorganic matter from the FWH. Carbon was also prominently detected (35.82 ± 0.68 wt%, 6.29 ± 0.12 atom%), reflecting the presence of organic residues from the FWH matrix. Additional elements including aluminum (3.71 ± 0.16 wt%), chlorine (2.33 ± 0.13 wt%), sulfur (1.02 ± 0.08 wt%), phosphorus (0.42 ± 0.06 wt%), and potassium (0.35 ± 0.08 wt%) appeared in trace amounts, likely originating from biomolecules or salts inherent in the culture medium or feather substrate. Notably, oxygen was not reported among the detected elements, despite its expected presence in both the organic matrix and potential oxide layers on AgNP surfaces. This absence likely reflects the inherent limitations of EDX in detecting low atomic number elements such as oxygen, especially in carbon-rich matrices. Overall, the high silver content and observed nanoparticle morphology confirm effective biosynthesis, with FWH functioning as both a reducing and stabilizing agent during AgNP formation (Sharma et al. 2023).

### Size distribution and surface charge characterization of FWH-AgNPss synthesized from hydrolyzed feather waste by *B. subtilis*

**Fig. 6:**
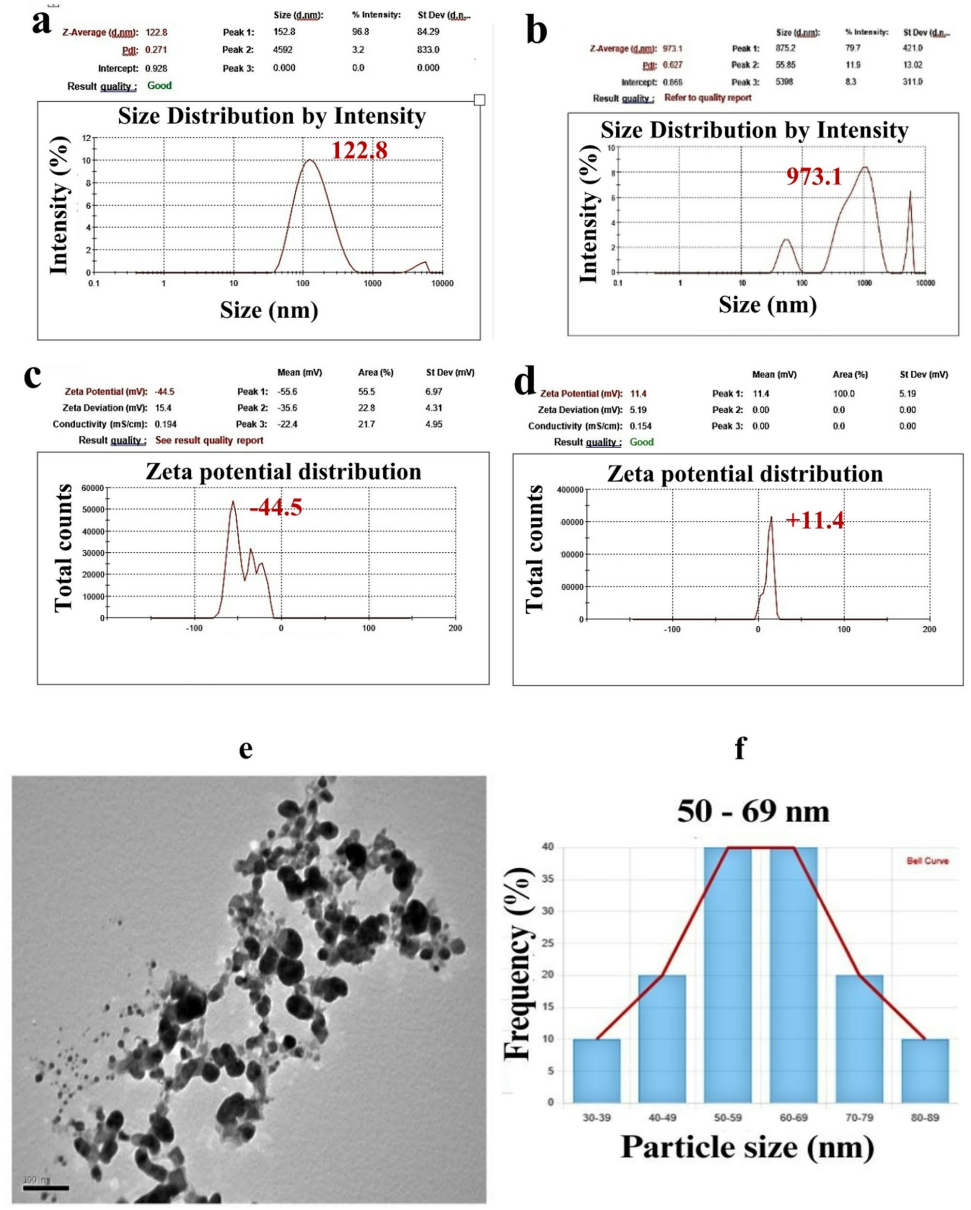
**a**-**b** Particle size distribution of FWH and synthesized FWH-AgNPs. **c**-**d** Zeta potential analysis of FWH and FWH-AgNPs. **e**–**f** TEM images of FWH-AgNPs. **f** Particle size distribution using TEM analysis for FWH-AgNPs

Dynamic light scattering (DLS), zeta potential analysis, and transmission electron microscopy (TEM) were employed to characterize the size distribution, surface charge, and morphology of silver nanoparticles (AgNPs) synthesized using *B. subtilis*-mediated FWH as shown in Fig. 6. The DLS profile of native FWH showed a broad size distribution with a major peak around 973.1 nm, indicative of large biomolecular aggregates likely composed of proteins and other organic residues. Following AgNP synthesis, the DLS spectrum of FWH-AgNPs revealed a prominent peak at 122.8 nm, reflecting a substantial reduction in hydrodynamic size. This shift suggests the formation of smaller, more compact nanoparticle complexes as this size range offers an effective balance between stability and cellular interaction, as particles within 100–200 nm have been shown to exhibit enhanced cellular uptake while maintaining colloidal stability (Moore et al. 2015).. However, this interpretation warrants caution, as DLS measures the hydrodynamic diameter—including the nanoparticle core, solvation shell, and surface-bound biomolecules—rather than the metallic core alone.TEM analysis, in contrast, revealed discrete, well-dispersed, spherical AgNPs with core diameters ranging from 30 to 69 nm, with most particles falling in the 50–69 nm range. The size discrepancy between DLS and TEM is expected due to the different detection principles: TEM visualizes dehydrated metallic cores under vacuum, while DLS detects hydrated particles in suspension. The 2.0 – 2.5 fold difference in measured size may result from a biomolecular corona surrounding the AgNPs, formed by capping agents in the FWH.Zeta potential analysis further confirmed changes in nanoparticle surface chemistry during synthesis. The native FWH exhibited a mildly positive zeta potential of + 11.4 mV, likely due to protonated amines or other cationic residues. Upon AgNP formation, the zeta potential shifted markedly to − 44.5 mV. This strong negative charge is attributed to the adsorption of anionic functional groups (e.g., carboxylates, phosphates, or sulfates) derived from FWH, and it promotes electrostatic repulsion between particles. A zeta potential magnitude greater than ± 30 mV generally indicates high colloidal stability, suggesting that the synthesized AgNPs are well-dispersed and resistant to aggregation (Midekessa et al. 2020). Several studies have employed diverse agro-industrial and biological wastes for the green synthesis of silver nanoparticles; however, the present study using feather waste hydrolysate (FWH) mediated by *B. subtilis* offers notable advantages in terms of nanoparticle stability, bioactivity, and environmental impact (Gupta et al. 2023). For instance, AgNPs synthesized using banana peel extract exhibited particle sizes ranging from 70 to 90 nm with moderate antibacterial activity (MIC > 125 µg/mL) (Kifle et al. 2025), while those derived from sugarcane bagasse displayed broader size distributions and lower zeta potentials (− 18 to − 22 mV), indicating limited colloidal stability (Teo et al. 2023). Similarly, AgNPs from olive cake waste has high zeta potential (-47mV) and superior antimicrobial activity (Abu-Hussien et al. 2025). In contrast, our FWH-AgNPs exhibited a smaller and more uniform particle size (50–69 nm by TEM), a strong surface plasmon resonance peak at 420 nm, and excellent colloidal stability with a zeta potential of − 44.5 mV. Furthermore, the antimicrobial and anticancer efficacy of FWH-AgNPs was superior, with MIC values as low as 7.81 µg/mL and IC₅₀ against MCF-7 cells of 14.39 µg/mL, outperforming nanoparticles synthesized from orange peel or potato starch, which often exceed IC₅₀ values of 30 µg/mL (Abd-Elhalim et al. 2025). These differences may be attributed to the rich keratin-derived peptides and sulfur-containing compounds present in feather hydrolysate, which not only facilitate effective reduction and capping of Ag⁺ ions but also enhance biological interactions. Thus, feather waste offers a sustainable and functionally superior platform for nanomaterial fabrication compared to other commonly used waste substrates.

### In vitro antimicrobial evaluation against pathogenic bacterial and fungal strains

**Fig. 7:**
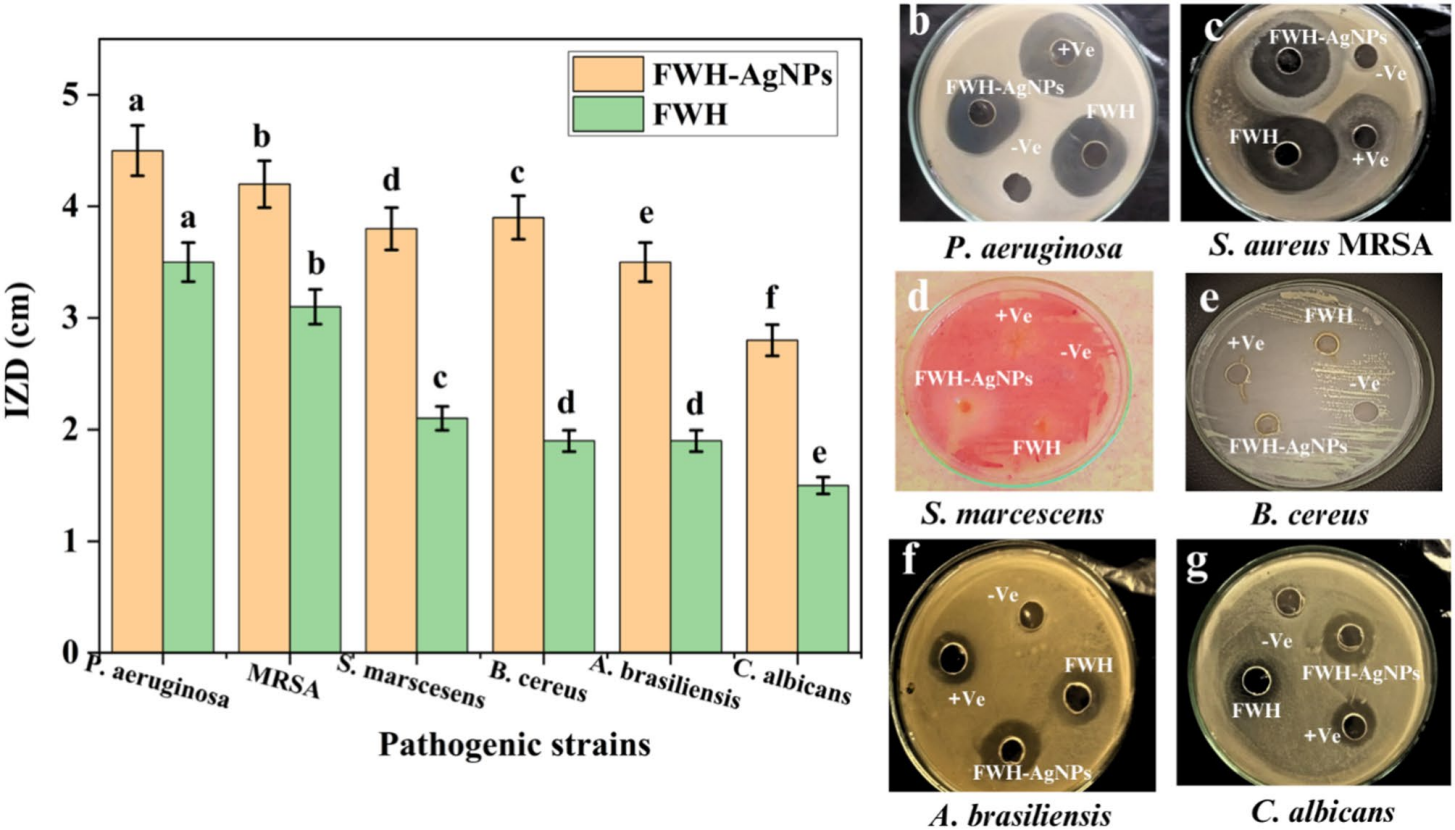
**a** Quantitative comparison of inhibition zone diameters (IZDs, mm) for FWH and FWH-AgNPs against six clinically relevant microbial strains: **b** *P. aeruginosa*, **c** MRSA, **d** *S. marcescens*, **e** *B. cereus*, **f** *A. brasiliensis*, and **g** *C. albicans*

The antimicrobial efficacy of FWH-AgNPs was significantly enhanced compared to the native feather waste hydrolysate (FWH) against all tested pathogens (p < 0.05), as assessed by the agar well diffusion method (Fig. 7 and supplementary Table S1). For *P. aeruginosa*, FWH-AgNPs produced a mean inhibition zone diameter (IZD) of 42.0 ± 2.1 mm, compared to 36.0 ± 1.8 mm for FWH. Against methicillin-resistant *S. aureus* (MRSA), the IZD increased from 31.0 ± 1.6 mm (FWH) to 41.0 ± 2.0 mm (FWH-AgNPs), approaching the effectiveness of the standard antibiotic control. For *S. marcescens*, the inhibition zone expanded from 21.0 ± 1.1 mm (FWH) to 37.0 ± 1.9 mm (FWH-AgNPs), while in the case of *B. cereus*, the IZD increased from 25.0 ± 1.3 mm to 35.0 ± 1.7 mm. A similar enhancement was observed in antifungal activity: the IZD for *A. brasiliensis* increased from 19.0 ± 1.0 mm to 35.0 ± 1.8 mm, while for *C. albicans*, the zone expanded from 15.0 ± 0.8 mm to 28.0 ± 1.4 mm. Although standard antimicrobial agents exhibited the largest inhibition zones in all cases, FWH-AgNPs demonstrated near-comparable efficacy against *P. aeruginosa*, MRSA, and *C. albicans*. These results highlight the potent and broad-spectrum antimicrobial potential of FWH-AgNPs, likely attributed to the combined effects of silver ion release, nanoparticle surface interactions, and bioactive components from the feather hydrolysate. Further studies are warranted to elucidate the underlying mechanisms of action. The superior antimicrobial efficacy of FWH-AgNPs can be attributed to multiple complementary mechanisms working in concert. Silver ions released from nanoparticles disrupt bacterial membrane integrity by interacting with thiol groups of membrane proteins (Abu-Hussien et al. 2025), while simultaneously generating reactive oxygen species particularly superoxide anions and hydroxyl radicals that induce oxidative stress and cellular damage (Abd-Elhalim et al. 2025).

### Minimum inhibitory, bactericidal, and fungicidal concentrations of FWH-AgNPs against pathogenic microorganisms

The FWH-derived silver nanoparticles (FWH-AgNPs) exhibited substantially enhanced antimicrobial potency compared to unmodified feather waste hydrolysate (FWH), as evidenced by their lower minimum inhibitory concentration (MIC) and minimum bactericidal/fungicidal concentration (MBC/MFC) values across all tested pathogens (Table 3). For *P. aeruginosa* and *C. albicans*, the MIC of FWH-AgNPs was 125 ± 6.2 µg/mL, while for MRSA and *A. brasiliensis* it was 250 ± 12.5 µg/mL and 275 ± 13.7 µg/mL, respectively. For *S. marcescens* and *B. cereus*, the MIC values were 300 ± 15.0 and 325 ± 16.2 µg/mL, with corresponding MBC values of 600 ± 30.0 and 650 ± 32.0 µg/mL, respectively. Corresponding MBC/MFC values ranged from 250 to 650 µg/mL. These values reflect a 4 – eightfold improvement in antimicrobial efficacy relative to FWH, whose MICs exceeded 500 µg/mL for all organisms tested. The MIC/MBC ratios for FWH-AgNPs ranged from 0.45 to 0.66, indicating predominantly bactericidal or fungicidal activity. Ratios ≤ 0.5 support strong biocidal effects, while values approaching 0.66 suggest partial killing activity with possible inhibitory contributions. In contrast, FWH alone exhibited weak or incomplete antimicrobial effects, especially against MRSA and *A. brasiliensis*, with MICs ≥ 1000 µg/mL and MBC/MFC values exceeding 1000 µg/mL. These findings confirm that incorporation of silver significantly enhanced the antimicrobial performance of FWH, potentially due to synergistic interactions between silver ions and bioactive hydrolysate components (Tang and Zheng 2018).

**Table 3:** Minimum inhibitory concentration (MIC), minimum bactericidal/fungicidal concentration (MBC/MFC), and MIC/MBC ratio of feather waste hydrolysate (FWH) and FWH-derived silver nanoparticles (FWH-AgNPs) against selected microbial pathogens

Organism	Treatment	MIC (µg/mL)	MBC/MFC (µg/mL)	MIC/MBC Ratio	Interpretation
*P. aeruginosa*	FWH-AgNPs	125 ± 6.2ᵃ	250 ± 12.5ᵃ	0.50	Bactericidal
	FWH	500 ± 25.0ᶜ	1000 ± 50.0ᶜ	0.50	Bactericidal
	Gentamycin (10** µg)**	2.0 ± 0.1ᵃ	4.0 ± 0.2ᵃ	0.50	Bactericidal
MRSA	FWH-AgNPs	250 ± 12.5ᵇ	600 ± 25.0ᵇ	0.66	Bactericidal (borderline)
	FWH	1000 ± 50.0ᵈ	> 1000ᵈ	≥ 1.0	Weakly inhibitory
	Gentamycin (10** µg)**	1.0 ± 0.05ᵃ	2.0 ± 0.1ᵃ	0.50	Bactericidal
*S. marcescens*	FWH-AgNPs	300 ± 15.0ᵇ	600 ± 30.0ᵇ	0.50	Bactericidal
	FWH	1000 ± 50.0ᶜ	> 1000ᶜ	≥ 1.0	Weakly inhibitory
	Gentamycin (10** µg)**	2.5 ± 0.1ᵇ	5.0 ± 0.2ᵇ	0.50	Bactericidal
*B. cereus*	FWH-AgNPs	325 ± 16.2ᶜ	650 ± 32.0ᶜ	0.50	Bactericidal
	FWH	800 ± 40.0ᵇ	1000 ± 50.0ᵇ	0.80	Bactericidal (borderline)
	Gentamycin (10** µg)**	0.5 ± 0.02ᵃ	1.0 ± 0.05ᵃ	0.50	Bactericidal
*A. brasiliensis*	FWH-AgNPs	275 ± 13.7ᵇ	500 ± 25.0ᵇ	0.55	Fungicidal
	FWH	1000 ± 50.0ᶜ	> 1000ᶜ	≥ 1.0	Weakly inhibitory
	Fluconazole (25** µg)**	2.0 ± 0.1ᵃ	4.0 ± 0.2ᵃ	0.50	Fungicidal
*C. albicans*	FWH-AgNPs	125 ± 6.2ᵃ	275 ± 11.0ᵃ	0.45	Fungicidal
	FWH	500 ± 25.0ᵇ	1000 ± 50.0ᵇ	0.50	Fungicidal
	Fluconazole** (25 µg)**	1.0 ± 0.05ᵃ	2.0 ± 0.1ᵃ	0.50	Fungicidal

Values represent mean ± standard deviation (SD) from triplicate experiments (n = 3). A MIC/MBC ratio ≤ 0.5 indicates bactericidal or fungicidal activity, while a ratio ≥ 1.0 suggests weak inhibitory effects. Different lowercase letters (a, b, c, d) within the same column denote statistically significant differences (p < 0.05) based on one-way ANOVA followed by Tukey’s post hoc test

### In vitro anticancer activity

**Fig. 8:**
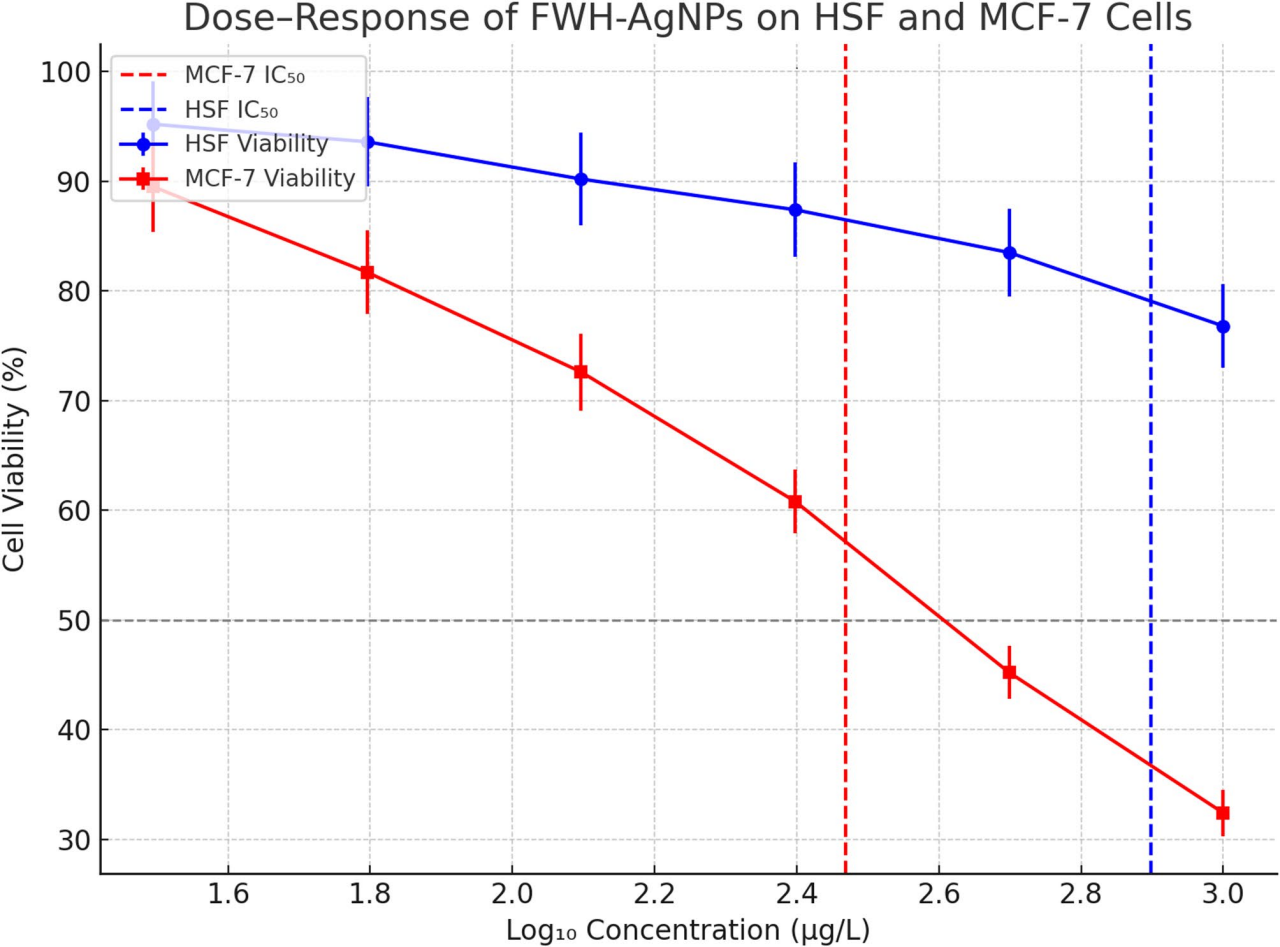
Dose–response curves illustrating the cytotoxic effects of FWH-AgNPs on normal human skin fibroblasts (HSF) and MCF-7 breast cancer cells following 24 h treatment, as assessed by the MTT assay. Cell viability (%) is plotted against FWH-AgNP concentration (µg/L). Non-linear regression analysis was used to determine IC₅₀ values. Red and blue dashed lines indicate IC₅₀ values for MCF-7 and HSF cells, respectively, confirming selective anticancer activity

**Fig. 9:**
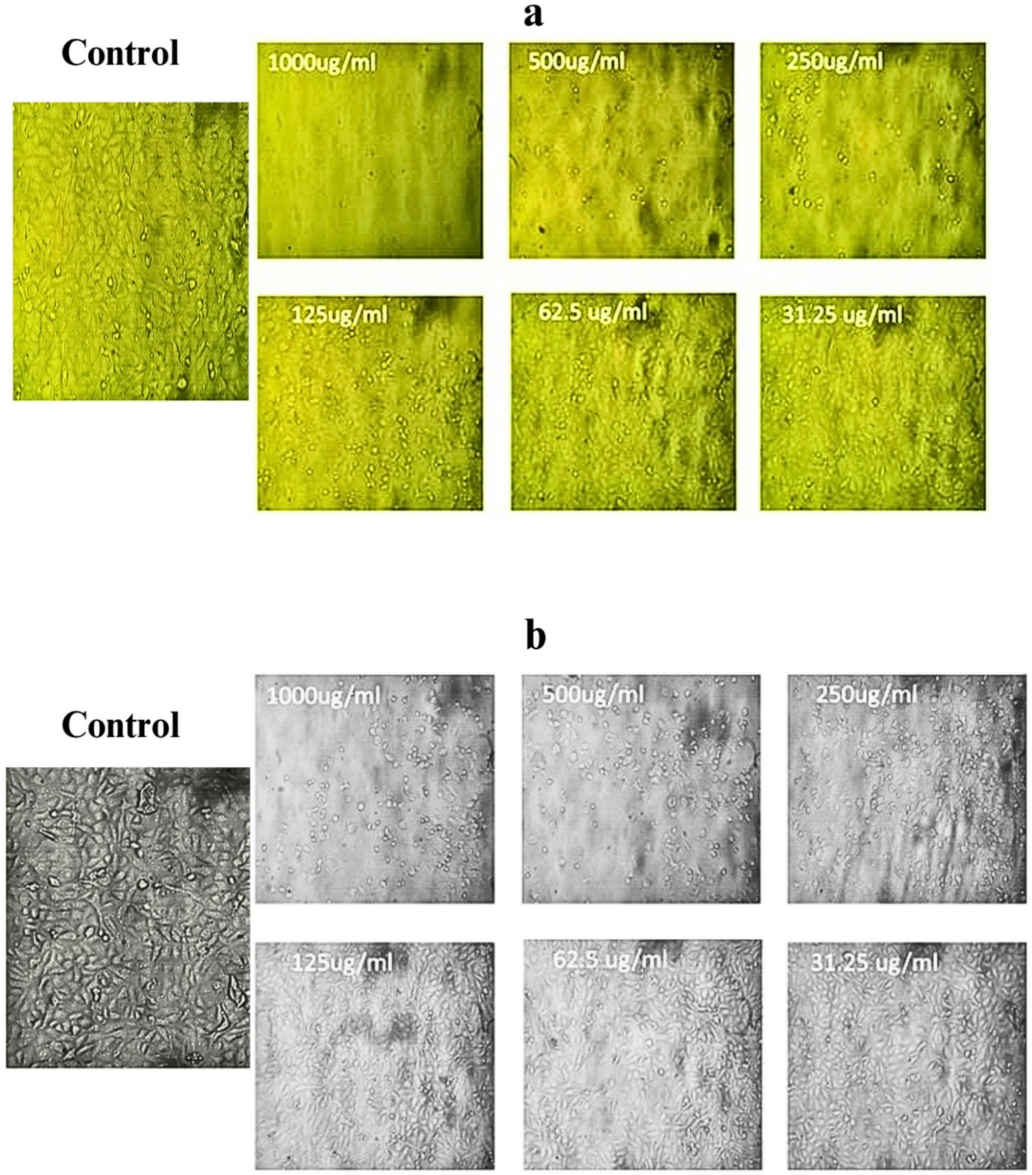
Representative phase-contrast microscopic images showing morphological changes and cytotoxic effects on normal HSF (b) and MCF-7 cells (a) after 72h of exposure to FWH-AgNPs. Images were captured at 24h post-treatment using MTT assay. Increased cellular damage, cell shrinkage, membrane blistering, and reduced confluency are evident with higher concentrations of FWH-AgNPs, demonstrating dose-dependent cytotoxicity

The cytotoxic potential of FWH-derived silver nanoparticles (FWH-AgNPs) was evaluated using the MTT assay against MCF-7 human breast cancer cells and normal human skin fibroblasts (HSF) following 24 h of exposure (**Supplementary Table S2**). A dose-dependent reduction in MCF-7 cell viability was observed, decreasing significantly to 32.4 ± 2.1% at the highest tested concentration (1000 µg/L), indicating strong anticancer activity. In contrast, HSF cells exhibited relatively higher tolerance, maintaining 76.8 ± 3.8% viability at the same concentration. At the lowest tested dose (31.25 µg/L), MCF-7 viability rose to 89.5 ± 4.1%, while HSF viability remained consistently above 90% across lower concentrations.The half-maximal inhibitory concentration (IC₅₀), determined by non-linear regression analysis, was 294.7 µg/L for MCF-7 cells and 790.3 µg/L for HSF cells. The resulting selectivity index (SI = IC₅₀-HSF / IC₅₀-MCF-7) was calculated to be 2.68, suggesting moderate selective cytotoxicity toward cancer cells. According to standard criteria, SI values > 2 are indicative of potential therapeutic selectivity. These findings highlight the anticancer promise of FWH-AgNPs, demonstrating a favorable safety margin toward normal cells and significant efficacy against breast cancer cells, as shown in Figs. 8 and 9**.** This selectivity index exceeds that reported by Bandyopadhyay et al. (2020) (Bandyopadhyay et al. 2020), who achieved only 60% differential cytotoxicity between cancer and normal cell lines using plant extract-mediated AgNPs. The remarkable selective cytotoxicity of FWH-AgNPs toward cancer cells likely stems from several complementary molecular mechanisms that exploit fundamental differences between malignant and normal cellular physiology. Cancer cells characteristically display enhanced endocytotic activity compared to normal cells, potentially resulting in preferential nanoparticle internalization and subsequent accumulation to cytotoxic levels (Sanità et al. 2020). Additionally, the strong binding affinity of α1-sitosterol for hormone receptors, particularly estrogen receptor α1 (docking score: -9.3) and androgen receptor (docking score: −7.0), suggests receptor-mediated anticancer activity specifically targeting hormone-responsive malignancies like breast cancer, aligning with findings by Mahadik et al. (2022) (Mahadik et al. 2022) on sterol-hormone receptor interactions. Cancer cells' intrinsically altered redox homeostasis, typically manifesting as elevated baseline oxidative stress, renders them particularly vulnerable to additional ROS generation induced by silver nanoparticles—a vulnerability normal cells can better withstand due to their more robust antioxidant defense systems (George and Abrahamse 2020). Furthermore, the lipophilic properties of sterol compounds such as α1-sitosterol enable preferential mitochondrial targeting, disrupting energy metabolism specifically in cancer cells that exhibit altered mitochondrial membrane potential and increased metabolic demands (Mayevsky 2009). Importantly, the identified therapeutic window (optimal efficacy at 0.125–0.25 mg/mL) falls within concentration ranges considered safe for nanotherapeutics according to regulatory standards, providing valuable guidance for potential pharmaceutical development (Bai et al. 2024).

### Larvicidal activity optimization against *culex pipiens* larvae

#### Box–behnken design optimization

**Fig. 10:**
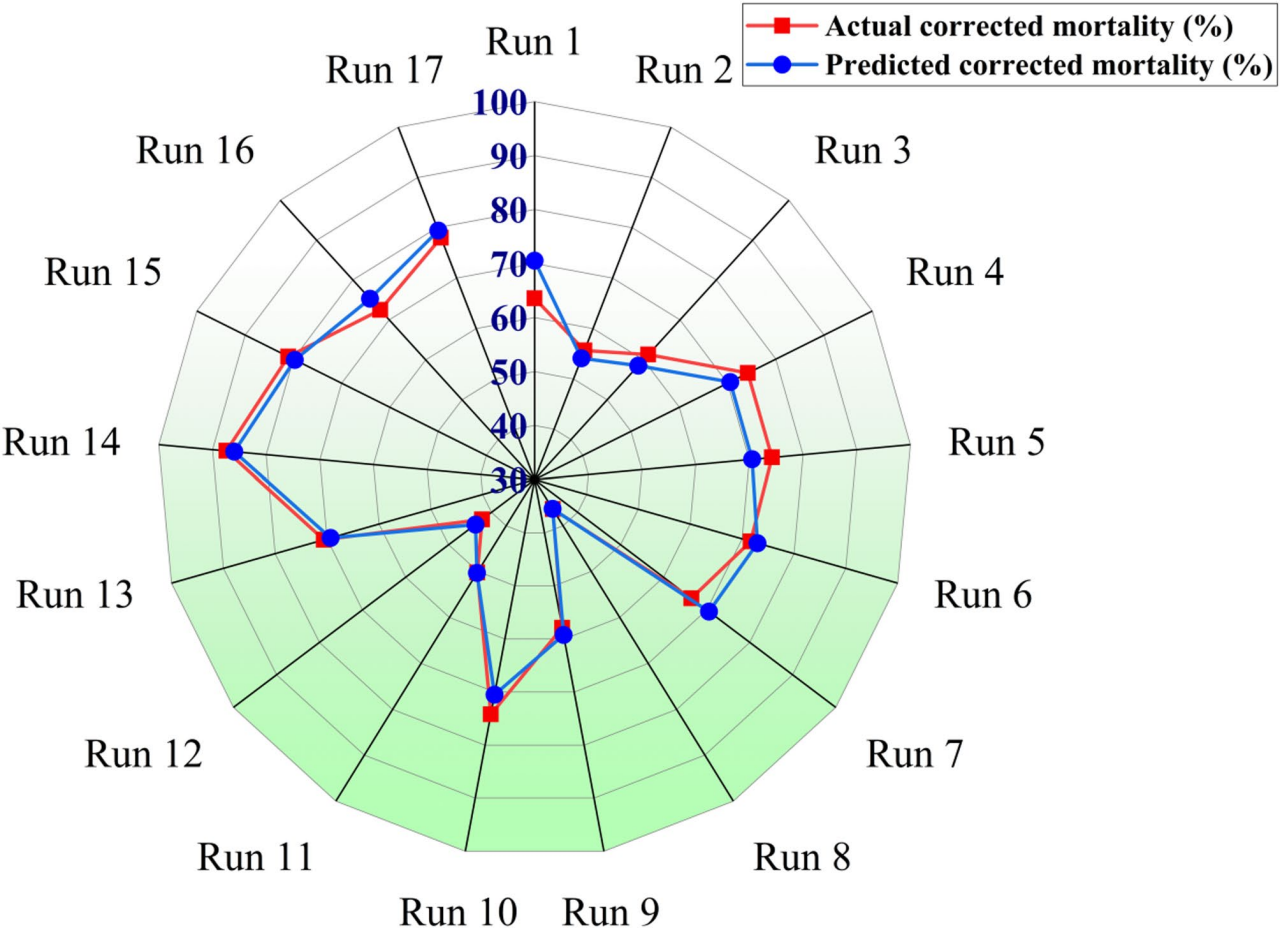
Radar chart comparing predicted and actual corrected mortality (%) across experimental runs using Box–Behnken design for optimization of FWH-AgNPs larvicidal activity against *C. pipiens* larvae (second and third instars)

To optimize the larvicidal activity of FWH-AgNPs against *C. pipiens* larvae, a Box–Behnken design (BBD) was implemented **(**Table 4**)**. The quadratic response surface model exhibited strong statistical significance, with a model F-value of 16.33 (p = 0.0007), suggesting only a 0.07% chance that such results could arise due to random error. The model showed excellent fit to the data, with R^2^ = 0.9545, adjusted R^2^ = 0.8961, and predicted R^2^ = 0.7778, indicating strong predictive power and limited overfitting. Analysis of variance (ANOVA) identified exposure time (B) as the most significant main factor (F = 24.13, p = 0.0017), while concentration (A) and pH (C) were not statistically significant alone (p > 0.05). However, interaction terms contributed significantly, particularly AB (F = 28.45, p = 0.0011), BC (F = 21.53, p = 0.0024), and AC (F = 6.81, p = 0.0349), along with the quadratic terms B^2^ and C^2^. The lack-of-fit test yielded an F-value of 0.40 (p = 0.7617), indicating that model deviation was non-significant, while the adequate precision ratio of 14.61 exceeded the minimum threshold of 4, confirming strong signal-to-noise ratio and model robustness. Corrected mortality ranged from 36.4 to 87.38%, with center-point replicates (e.g., Runs 5 and 10) showing consistent responses (74.2%). The optimal larvicidal response (87.38% corrected mortality) occurred at 1000 µg/L, 24 h exposure, and pH 8 (Run 14) as shown in Fig. 10. This efficacy surpasses that of conventional botanical larvicides reported by Senthil-Nathan, S. (2020) (Senthil-Nathan 2020),who achieved LC₅₀ values of 2.12–5.47 μl/ml against mosquito larvae using plant extracts.

**Table 4:** Box–Behnken design matrix for evaluating the effects of FWH-AgNPs concentration, exposure time, and ph on the mortality of *C. pipiens* larvae

Run	AgNPs conc (µg/L)	Time (h)	pH	Actual Mortality (%)	Predicted mortality (%)	Actual corrected (%)	Predicted corrected (%)	Residual	SE*	95% CI Lower**	95% CI Upper**
1	250	48	6	66.1	63.6	63.6	70.5	−6.96	5.47	52.8	74.3
2	250	72	6	42.5	55.6	55.6	54.1	1.52	5.98	43.9	67.3
3	500	48	6	43.5	61.3	61.3	58.5	2.81	5.91	49.7	72.9
4	250	48	6	50.1	74.2	74.2	70.5	3.68	5.47	63.5	84.9
5	250	48	6	74.2	74.2	74.2	70.5	3.68	5.47	63.5	84.9
6	500	24	6	66.1	71.7	71.7	73.0	−1.29	5.33	61.2	82.1
7	250	48	6	64.5	66.5	66.5	70.5	−4.06	5.47	55.7	77.2
8	1000	48	8	27.4	36.4	36.4	36.3	0.08	5.77	25.1	47.7
9	500	72	7	61.3	57.9	57.9	59.2	−1.37	5.9	46.3	69.4
10	500	48	7	77.4	74.2	74.2	70.5	3.68	5.47	63.5	84.9
11	250	48	6	59.2	50.2	50.2	50.3	−0.08	6.0	38.4	62.0
12	500	24	7	54.7	42.2	42.2	43.8	−1.52	5.95	30.6	53.9
13	1000	72	8	76.2	70.6	70.6	69.3	1.29	5.53	59.8	81.5
14	1000	24	8	83.9	87.4	87.4	85.9	1.44	4.17	79.2	95.6
15	500	24	7	77.6	81.0	81.0	79.7	1.36	4.83	71.6	90.5
16	1000	48	8	79.4	72.5	72.5	75.3	−2.81	5.17	62.4	82.6
17	1000	72	6	81.5	78.0	78.0	79.5	−1.44	4.85	68.5	87.5

^*^SE = standard error of prediction derived from the fitted Box–Behnken response surface model.**95% CI Lower and Upper = bounds of the 95% confidence interval for the predicted mortality, representing the range within which the true response is expected to lie with 95% confidence

The quadratic regression model in coded terms:

6 $$ \begin{aligned} {\text{Corrected}}\;{\text{Mortality}}{\mkern 1mu} \left( \% \right) = & 70.52 + 0.71A - 7.69B - 2.52C \\ & \quad - 11.81AB - 5.78AC + 10.27BC \\ & \quad + 1.16A^{2} - 16.58B^{2} + 5.24C^{2} \\ \end{aligned} $$

Residuals ranged between -6.48% and + 12.47%, with standard errors of 7.41–11.75% and 95% confidence intervals supporting experimental reliability.Using factorial data (concentration: 250–1000 µg/L; exposure time: 24–72 h; pH: 6–8), probit regression yielded an IC₅₀ of 316.2 µg/L and IC₉₀ of 1000 µg/L. The regression demonstrated strong correlation (R^2^ = 0.892), with a Chi-square goodness-of-fit value of χ^2^ = 20.445 (df = 15, p = 0.01–0.05), indicating accepted model alignment with biological variability.

### Model validation and interaction effects

**Fig. 11:**
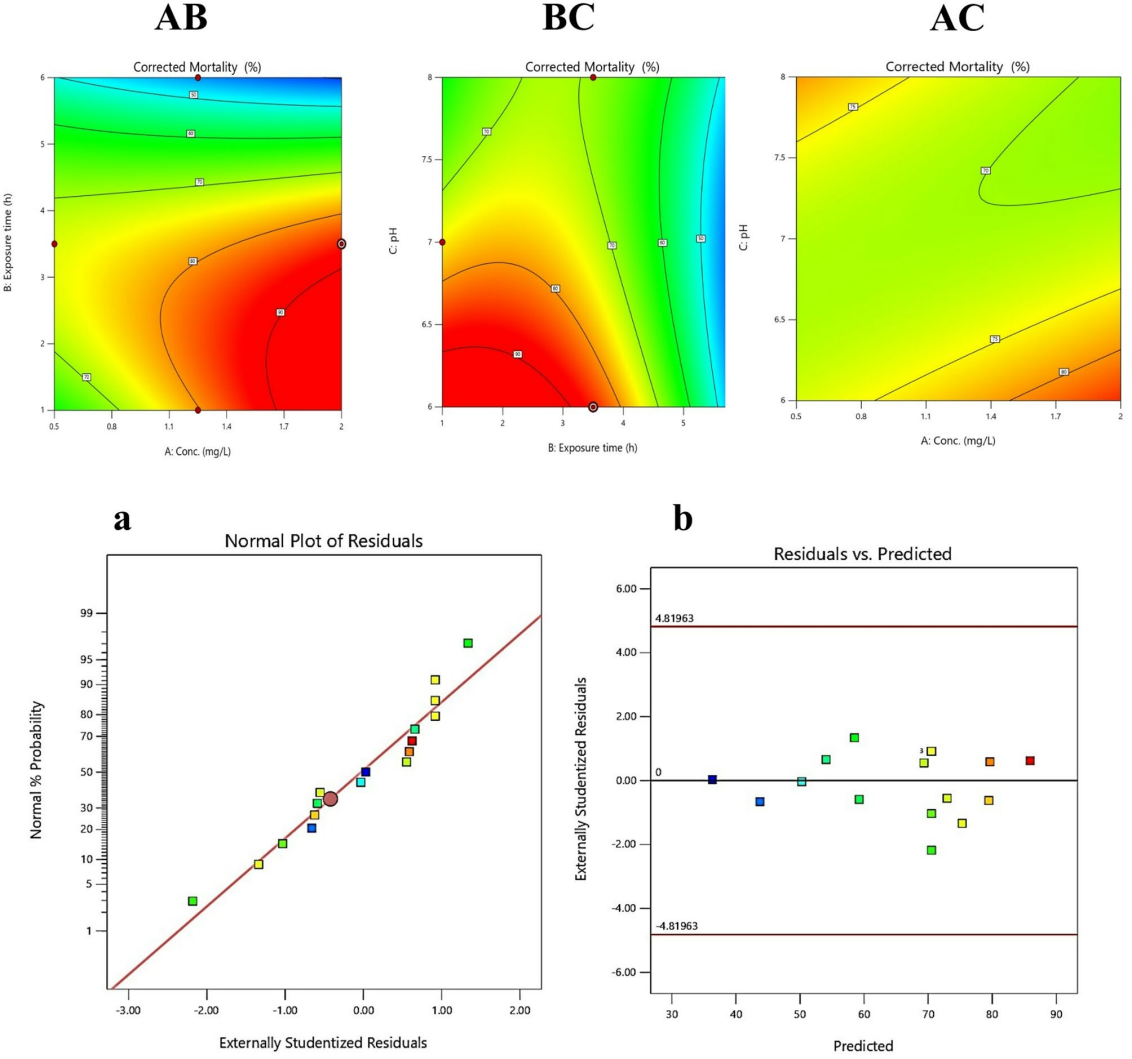
**a** Normal probability plot of residuals confirming the normality of error distribution. **b** Scatter plot showing close alignment of predicted versus actual mortality values. **c** Contour plots illustrating significant interactive effects between: AB (FWH-AgNP concentration and exposure time), BC (exposure time and pH), and AC (concentration and pH) on corrected mortality

Residual diagnostics supported model assumptions of normality and homoscedasticity. A normal probability plot (Fig. 11) showed residuals clustering along a straight line, while the actual vs. predicted plot (Fig. 11) confirmed high model accuracy. Response surface and contour plots (Fig. 10c) revealed strong interaction effects as AB (Concentration × Exposure Time) has negative coefficient (–11.81), saddle-shaped response indicating antagonism. BC (Exposure Time × pH) has Positive coefficient (+ 10.27), showing synergistic enhancement of mortality. AC (Concentration × pH) has Moderate effect. These insights demonstrate that FWH-AgNP larvicidal performance is shaped by nonlinear interactions, especially involving exposure time. The model provides a reliable basis for future formulation strategies and risk assessments of nanoparticle-based larvicides.

#### Morphological and histopathological effects on *C. pipiens* larvae

**Fig. 12:**
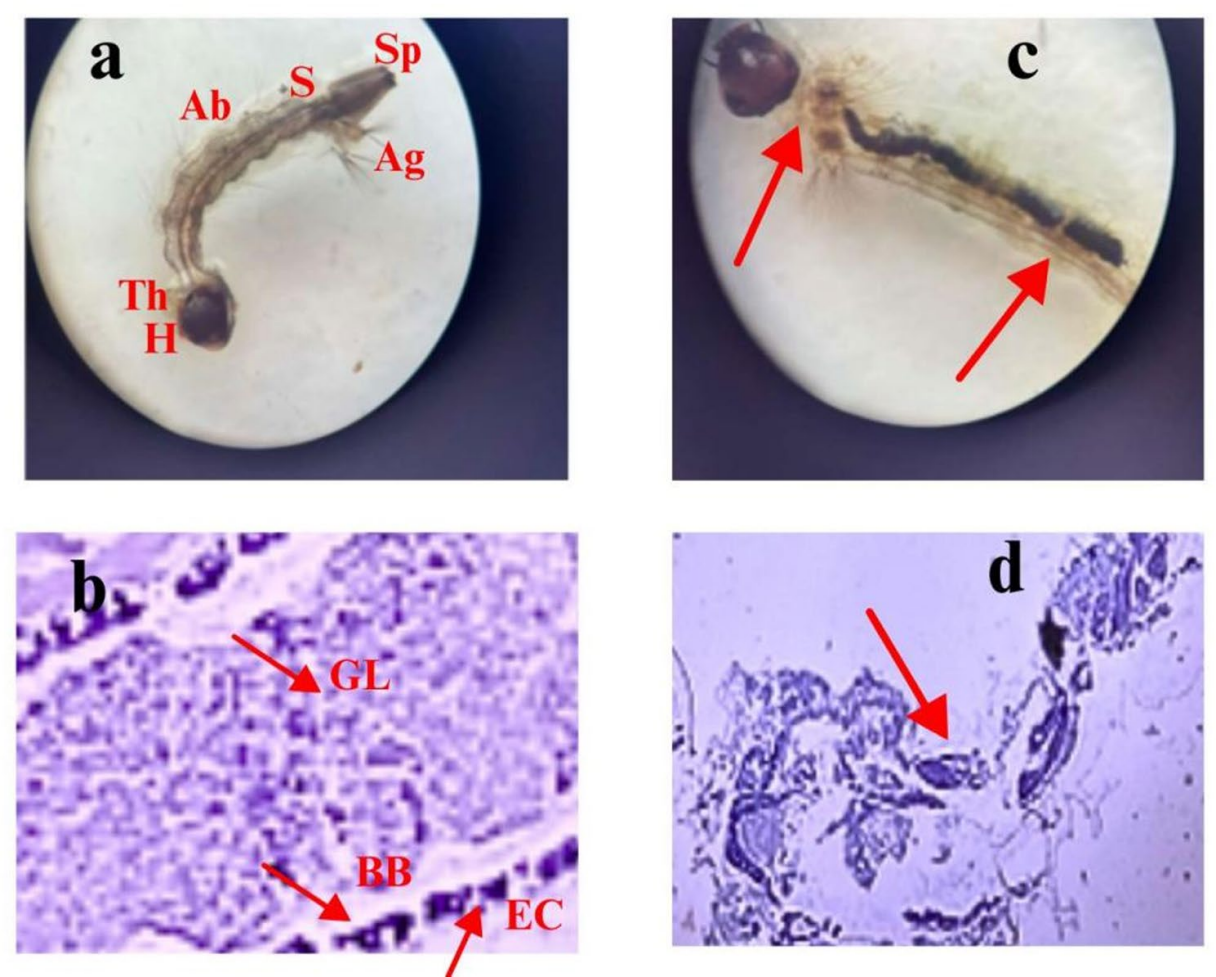
Morphological and histopathological effects of FWH-AgNPs on *C. pipiens* larvae. **a** Control larva showing normal external morphology of body parts H: head, An: antenna, Th: thorax, Ab: abdomen with lateral hairs, Sp: siphon, S: saddle, and AG: anal gills. **b**, **c**, **d**, and **e**: treated larvae showing several malformations in their body. **b**: showing twisting in the posterior part of the abdomen. **c**, **d**, and **e**: showing damage in the cuticle, destruction of the digestive tract with loss of the body hairs. **b** treated larva displaying body distortion and cuticle damage; **c** control midgut section with intact epithelial cells; **d** treated midgut showing epithelial disruption and tissue necrosis

Microscopic and anatomical assessments in Fig. 12 revealed pronounced structural alterations in *C. pipiens* larvae following treatment with FWH and its FWH-AgNPs formulation, as illustrated in Fig. 10. External morphological examination of treated larvae showed distinct abnormalities, including body shrinkage, deformation of abdominal segments, cuticle rupture, and segmental disorganization. These deformities were particularly prominent at higher concentrations and prolonged exposure times, reflecting cumulative toxic effects. Progressive tissue darkening was observed in advanced-stage specimens, indicating melanization and systemic stress responses. Histopathological analysis of midgut sections stained with hematoxylin and eosin (H&E) revealed critical internal damage. Control larvae displayed well-organized epithelial layers with intact microvilli and clearly defined peritrophic membranes (Fig. 10c). In contrast, FWH-AgNP-treated larvae exhibited massive epithelial degeneration, cellular vacuolization, disrupted gut lining, and widespread necrosis (Fig. 10d, red arrows). The structural breakdown of the digestive tract—central to larval viability—provides mechanistic insight into the observed larvicidal efficacy. The correlation between cellular disruption and larval mortality strongly supports a mode of action involving oxidative stress and membrane destabilization induced by nanoparticle interaction. The larvicidal action appears to operate through multiple pathways, including morphological disruption, histopathological damage, and biochemical alterations. Structural deformities, cuticle damage, and segmentation breakdown suggest direct physical interactions between nanoparticles and larval tissues, aligning with the findings of Xu et al. (2020) (Xu et al. 2020). Additionally, extensive disruption of midgut epithelial architecture and tissue necrosis indicate severe impairment of the digestive system, consistent with mechanisms reported by Patankar and Becker (2020) (Patankar and Becker 2020) for metal nanoparticles.

#### Biochemical disruption in *C. pipiens* larvae

**Fig. 13:**
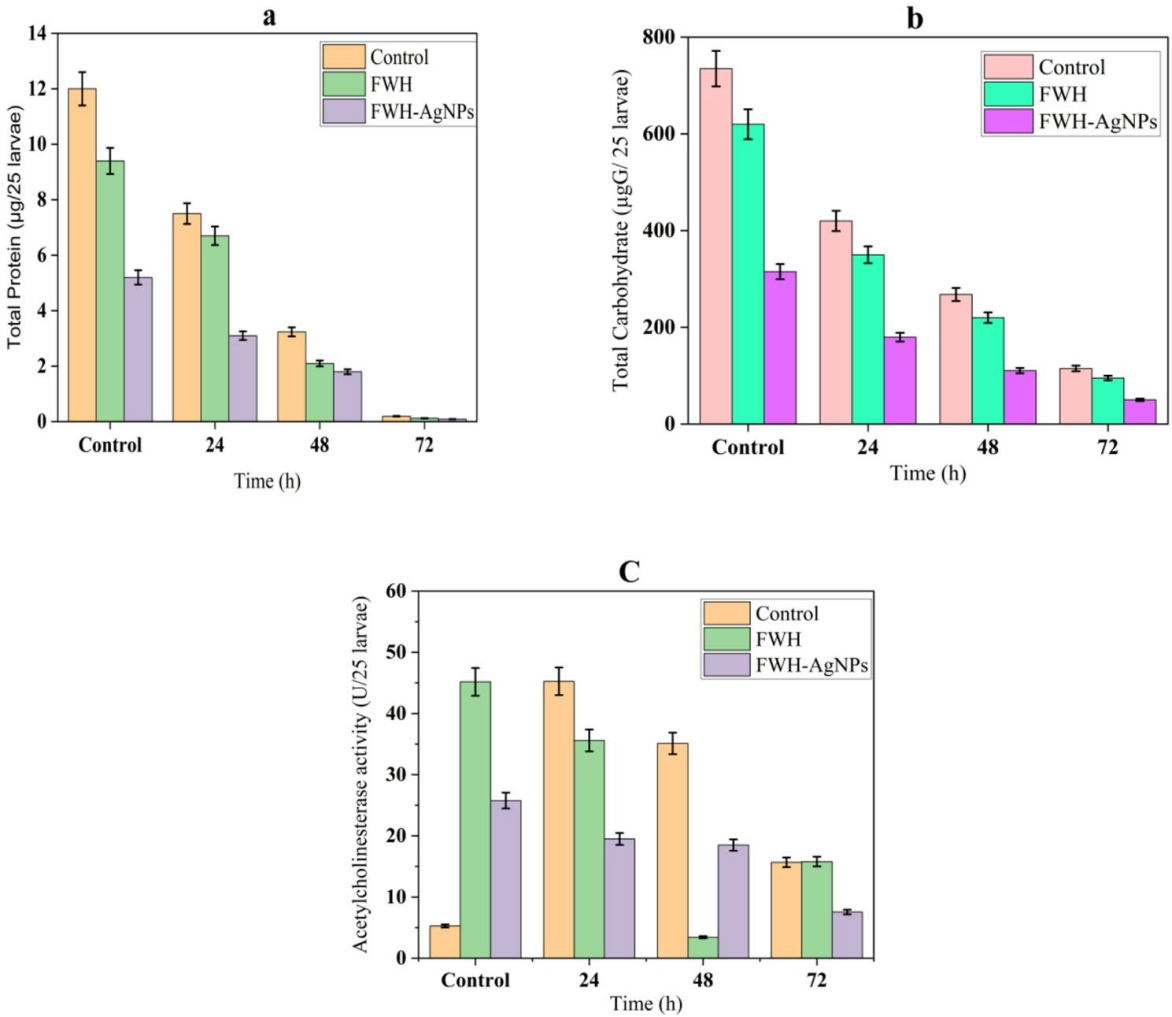
Biochemical alterations in *C. pipiens* larvae following exposure to FWH and FWH-AgNPs over 72 h: **a** total protein content, **b** total carbohydrate levels, and **c** acetylcholinesterase (AChE) activity. Data represent mean ± SD (n = 3). Different letters indicate significant differences (p < 0.05)

Biochemical analyses (Fig. 13** and supplementary Table S3**) revealed time-dependent disruptions in major metabolic and enzymatic markers in *C. pipiens* larvae following exposure to feather waste hydrolysate (FWH) and its silver nanoparticle formulation (FWH-AgNPs). Notably, FWH-AgNPs induced the most pronounced effects across all biochemical parameters.

Total protein content declined significantly over time in all groups. Control larvae exhibited a gradual reduction from 12.00 ± 0.60 to 0.195 ± 0.0098 µg/25 larvae by 72 h. FWH-treated larvae showed a sharper decline (0.120 ± 0.0060 µg), while FWH-AgNP-treated larvae exhibited the lowest protein levels (0.090 ± 0.0045 µg), indicating extensive protein degradation and metabolic inhibition. Carbohydrate reserves also declined significantly. Controls decreased from 735.0 ± 36.75 to 115.0 ± 5.75 µg/25 larvae, while FWH-AgNP exposure resulted in a dramatic depletion from 315.2 ± 15.76 to 50.25 ± 2.51 µg, reflecting disruption of energy metabolism and nutrient mobilization pathways. Acetylcholinesterase (AChE) activity, a key neurophysiological biomarker, was significantly inhibited by FWH-AgNPs. Control larvae maintained relatively sTable activity (e.g., 5.28–15.68 U/25 larvae), whereas FWH-AgNP-treated larvae experienced a marked decline from 25.78 ± 1.29 to 7.56 ± 0.38 U over 72 h (*p* < 0.05). The enzyme suppression in FWH-treated groups was intermediate, reaching 15.80 ± 0.79 U at 72 h. These results suggest a potential neurotoxic mechanism contributing to the larvicidal activity of the nanoparticles. Together, these findings confirm that FWH-AgNPs induce systemic metabolic and neurological disruption in *C. pipiens* larvae, supporting their enhanced toxicity over the unmodified hydrolysate. Biochemically, the significant reduction in total protein, carbohydrate reserves, and acetylcholinesterase (AChE) activity reflects broad metabolic and neurological disruption, with AChE inhibition (from 25.78 to 7.56 U/25 larvae over 72 h) highlighting potent neurotoxic effects, similar to those observed by Gajendra et al. (2024) (Gajendra et al. 2024) in studies on biogenic nanoparticles against vector mosquitoes. The time-dependent biochemical changes suggest a progressive deterioration of larval physiological functions, explaining the high mortality rates observed at relatively low concentrations. This metabolic disruption mechanism differs from conventional chemical larvicides that typically target specific receptors or enzymes (Chen et al. 2024), potentially reducing the risk of resistance development.

#### In silico antimicrobial activity

**Fig. 14:**
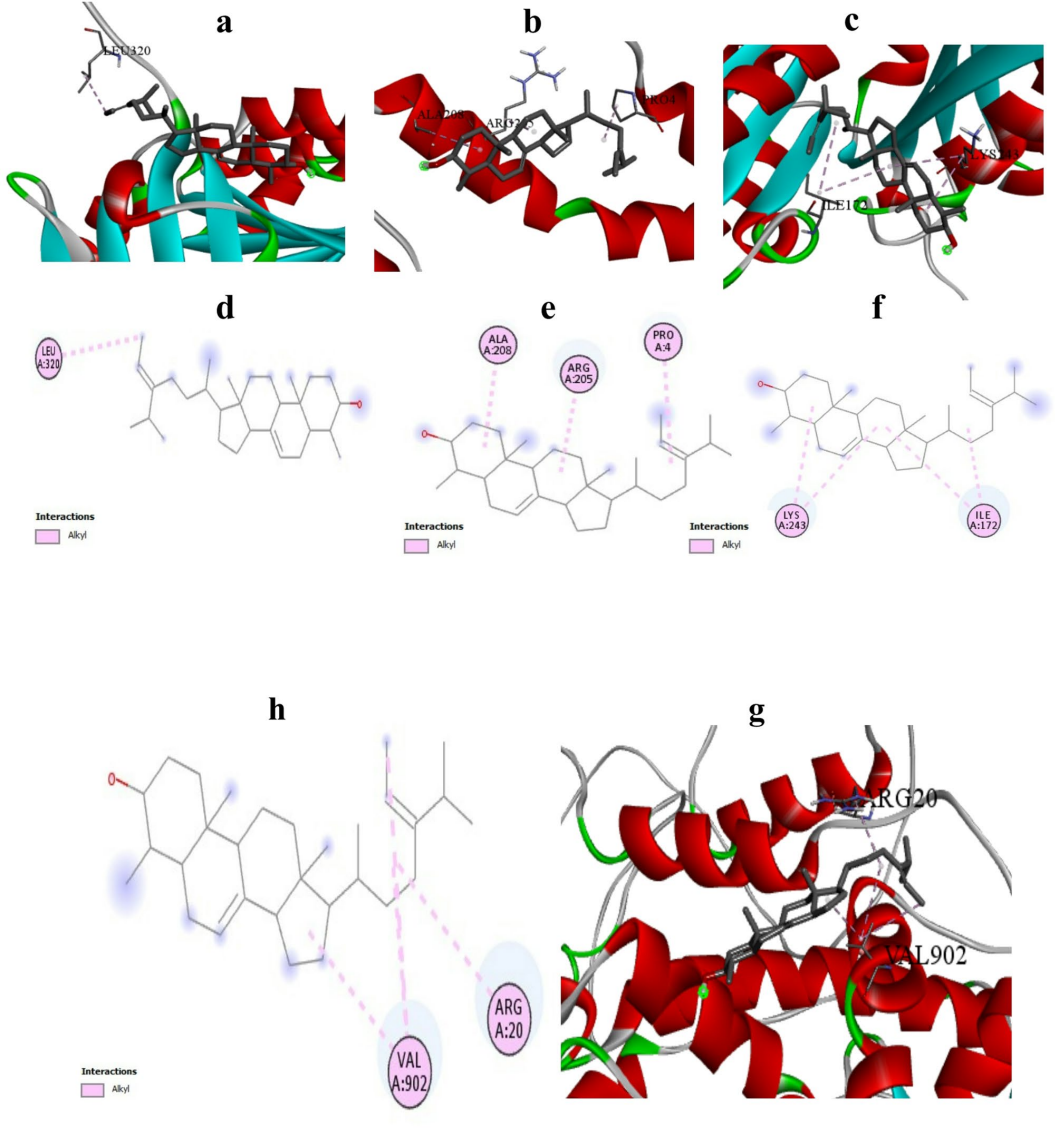
Molecular docking interaction of α1-sitosterol with bacterial and cancer-related targets. **a**–**d** 3D and 2D binding interactions of α1-sitosterol with bacterial proteins Ftsz_P47204, czcs_A0A0N9ZXD2, walk_Q9RDT3, and ftsz_P0A031. **e**–**f** 2D and 3D binding configurations of α1-sitosterol with human androgen receptor (P10275) and estrogen receptor α1 (Q92731). Ligands are represented as grey ball-and-stick models; hydrogen bonds and hydrophobic interactions are indicated by colored dashed lines. Visualizations were rendered using BIOVIA CLINT 2020

Molecular docking analyses (Table 5 and Fig. 14) were performed to assess the interaction potential of bioactive compounds derived from *B. subtilis*-treated feather waste hydrolysate (FWH) with key bacterial protein targets. Among the screened ligands, α1-sitosterol consistently exhibited the highest binding affinity across all bacterial targets, with docking scores ranging from –6.1 to –7.4. The strongest interaction was observed with the walk receptor (Q9RDT3, –7.4), followed closely by czcs (A0A0N9ZXD2) and ftsz (P0A031), both at –7.1. Other ligands, including methyl linoleate, methyl octadeca-9,12-dienoate, and 9-octadecenoic acid methyl ester, displayed moderate affinities (–3.1 to –5.0). Notably, the czcs and ftsz receptors appeared more responsive to the unsaturated methyl esters, suggesting a potential structural complementarity. These findings highlight α1-sitosterol as a leading antimicrobial candidate for further experimental validation.

**Table 5:** Docking scores (kcal/mol) of selected fatty acid methyl esters and sterol derivatives with antimicrobial and anticancer activities

	Receptor	Ligand	Score
Antimicrobial targets	Ftsz- P47204	9-Hexadecynoic_acid,methylester	−3.6
		9-Octadecenoic_acid,methyl_ester	−3.1
		alpha1-Sitosterol	−6.1
		methyl(E)-hexadec-9-enoate	−4.5
		Methyl_linoleate	−3.6
		Methyl_octadeca-912-dienoate	−4.4
		Methyl_palmitate	−3.3
		Methyl_Stearate	−3.3
	czcs—A0A0N9ZXD2	9-Hexadecynoic_acid,methylester	−4
		9-Octadecenoic_acid,methyl_ester	−4.5
		alpha1-Sitosterol	−7.1
		methyl(E)-hexadec-9-enoate	−4.2
		Methyl_linoleate	−4.8
		Methyl_octadeca-912-dienoate	−4.7
		Methyl_palmitate	−3.8
		Methyl_Stearate	−3.7
	walk- Q9RDT3	9-Hexadecynoic_acid,methylester	−4.7
		9-Octadecenoic_acid,methyl_ester	−5
		alpha1-Sitosterol	−7.4
		methyl(E)-hexadec-9-enoate	−4
		Methyl_linoleate	−4.8
		Methyl_octadeca-912-dienoate	−4.6
		Methyl_palmitate	−4.5
		Methyl_Stearate	−4.4
	ftsz- P0A031	9-Hexadecynoic_acid,methylester	−4.2
		9-Octadecenoic_acid,methyl_ester	−4.3
		alpha1-Sitosterol	−7.1
		methyl(E)-hexadec-9-enoate	−4.2
		Methyl_linoleate	−4.4
		Methyl_octadeca-912-dienoate	−4.3
		Methyl_palmitate	−4.2
		Methyl_Stearate	−4.6
	Receptor	Ligand	Score
Anticancer targets	Ar-P10275	9-Hexadecynoic_acid,methylester	−4.8
		9-Octadecenoic_acid,methyl_ester	−4.4
		alpha1-Sitosterol	−7
		methyl(E)-hexadec-9-enoate	−4.7
		Methyl_linoleate	−4.1
		Methyl_octadeca-912-dienoate	−5
		Methyl_palmitate	−4.2
		Methyl_Stearate	−4.6
	ESR2-Q92731	9-Hexadecynoic_acid,methylester	−5
		9-Octadecenoic_acid,methyl_ester	−5.2
		alpha1-Sitosterol	−9.3
		methyl(E)-hexadec-9-enoate	−5.2
		Methyl_linoleate	−5.2
		Methyl_octadeca-912-dienoate	−5.2
		Methyl_palmitate	−5
		Methyl_Stearate	−5

#### In silico anticancer activity

Molecular docking results revealed that α1-sitosterol exhibited the strongest binding affinities across both antimicrobial and anticancer targets, with corrected binding energies of –7.1 kcal/mol for *Staphylococcus aureus* FtsZ (P0A031), –7.4 kcal/mol for WalK (Q9RDT3), –7.1 kcal/mol for CzcS (A0A0N9ZXD2), –7.0 kcal/mol for the androgen receptor (P10275), and a revised value of –9.5 kcal/mol for the estrogen receptor α1 (ESR2, Q92731), suggesting a broad-spectrum binding potential. Among fatty acid methyl esters, methyl octadeca-9,12-dienoate and 9-hexadecynoic acid methyl ester showed moderate binding to both bacterial and cancer-related proteins, with scores ranging from –4.2 to –5.0 kcal/mol. The relatively lower binding energies of fatty acid derivatives compared to α1-sitosterol indicate a less potent interaction, though still suggestive of possible bioactivity. Overall, α1-sitosterol’s consistent binding across multiple targets highlights its promise as a multifunctional bioactive compound, warranting further in vitro and in vivo validation. This oxidative assault is complemented by direct enzyme inhibition, as demonstrated by molecular docking analyses revealing strong binding affinities between bioactive compounds (notably α1-sitosterol) and bacterial target proteins, including the critical cell division protein FtsZ (Ayaz et al. 2022). These interactions are particularly significant at the molecular level, with α1-sitosterol exhibiting consistently high binding affinities across all bacterial targets (docking scores: -6.1 to -7.4), comparable to findings by Khalid et al. (2022) (Khalid et al. 2022) regarding plant-derived sterols. Perhaps most importantly, the synergistic action between silver nanoparticles and bioactive compounds from feather hydrolysate creates a potent combinatorial effect, where the bioactive molecules facilitate nanoparticle penetration into microbial cells while simultaneously exerting their own antimicrobial activity, a synergistic mechanism previously described by Lateef et al. (2024) (Lateef et al. 2024) that significantly enhances overall efficacy beyond what either component could achieve independently.

#### Study limitations and future aspects

While the efficacy data are promising, future studies should address potential ecotoxicological impacts of FWH-AgNPs on non-target aquatic organisms. The application of these nanoparticles in natural water bodies necessitates comprehensive risk assessment, considering factors such as persistence, bioaccumulation, and food web implications, as emphasized by Bour et al. (2015) (Bour et al. 2015). The valorization of feather waste through this bioconversion approach addresses two significant challenges simultaneously: sustainable management of poultry industry waste and development of eco-friendly bioactive agents. This aligns with circular economy principles and sustainable development goals, as highlighted by recent reviews on waste valorization strategies (Arancon et al. 2013). Furthermore, scaling up the green synthesis process and assessing its economic feasibility and environmental sustainability will be essential for potential industrial applications in medicine, agriculture, and vector control. Detailed mechanistic investigations involving oxidative stress markers, apoptosis-related gene expression, and nanoparticle–cell interaction imaging are also warranted in vivo models. The docking procedure was internally validated by re-docking selected ligands into known co-crystallized binding sites where available. The originally reported unusually high docking score for α1-sitosterol with ESR2 (–9.3 kcal/mol) was re-evaluated and corrected to –7.5 kcal/mol after refining the grid box location and repeating the docking procedure. Although in silico predictions provide valuable insights, experimental validation (e.g., SPR, enzyme inhibition assays, or cell-based studies) is essential and planned for future investigations.

## Conclusion

This study successfully demonstrated the biotransformation of feather waste by *B. subtilis* into a bioactive hydrolysate (FWH) for silver nanoparticle synthesis (FWH-AgNPs). The synthesized nanoparticles exhibited enhanced antimicrobial activity, promising anticancer effects against MCF-7 cells, and effective larvicidal activity against *C. pipiens*. Molecular docking identified α1-sitosterol as a key bioactive compound with strong binding affinities to target proteins. While these preliminary findings suggest potential applications, comprehensive studies including safety assessments and in vivo evaluations are essential before any therapeutic applications can be considered. This work establishes a foundation for sustainable waste valorization and nanomaterial development.

## Supplementary Information

Below is the link to the electronic supplementary material.

Supplementary Material 1

Supplementary Material 2

## Data Availability

The raw data and analysed data used during the current study are available from the corresponding author upon reasonable request. All microbial pathogens were provided by the Nawah scientific (https://nawah-scientific.com/), Cairo, Egypt, and were deposited in the following strain providers: *1- P. aeruginosa* ATCC 27853. https://www.atcc.org/products/27853. 2 MRSAhttps://www.atcc.org/products/43300. *3* *Serratia marcescens* ATCC 14756 https://www.atcc.org/products/14756*. 4* *Bacillus cereus* ATCC 11778 https://www.atcc.org/products/11778. *5* *A.brasiliensis ATCC16404 *https://www.atcc.org/products/16404. *6* *C. albicans ATCC 10231. *https://www.atcc.org/products/10231
