# Supplementary material for: Green synthesis of silver nanoparticles from Bacillus subtilis-mediated feather hydrolysate: antimicrobial, larvicidal against culex pipiens, and anticancer activities
Source: Bioresour Bioprocess. 2025 Oct 17;12(1):116. doi: 10.1186/s40643-025-00952-y (PMC12534213; doi:10.1186/s40643-025-00952-y)
Supplement: Supplementary file 2 — Supplementary Material 2 [file 40643_2025_952_MOESM2_ESM.docx]

**Green synthesis of silver nanoparticles from *Bacillus subtilis*-mediated feather hydrolysate: Antimicrobial, Larvicidal against *Culex pipiens*, and Anticancer activities**

**Mohammed H. Alruhaili^1,2^, Samy Selim^3*^, Eslam Adly^4^,Mohanned T. Alharbi^5^, Bassam M. Al-ahmadi^6^, Mutasem S. Almehayawi^7^, Soad K. Al Jaouni^8^, Salem S. Salem^9^,Samah H. Abu-Hussien^10^**

^1^Department of Clinical Microbiology and Immunology, Faculty of Medicine, King Abdulaziz University, 21589, Jeddah, Saudi Arabia

^2^Special Infectious Agents Unit, King Fahad Medical Research Center, King AbdulAziz University, Jeddah, Saudi Arabia

^3^Department of Clinical Laboratory Sciences, College of Applied Medical Sciences, Jouf University, 72388, Sakaka, Saudi Arabia

^4^Department of Zoology, Faculty of Science, Ain Shams University, Cairo, Egypt

^5^Department of Basic Medical Sciences, College of Medicine, University of Jeddah, Jeddah, Saudi Arabia

^6^Department of Biology, College of Science, Taibah University, Madinah, 42353, Saudi Arabia

^7^Department of Emergency Medicine, Faculty of Medicine, King Abdulaziz University, Jeddah, Kingdom of Saudi Arabia

^8^Department of Hematology/Oncology, Chair of Prophetic Medicine Application, Faculty of Medicine, King Abdulaziz University, 21589, Jeddah, Saudi Arabia

^9^Botany and Microbiology Department, Faculty of Science, Al-Azhar University, Nasr City, Cairo-11884, Egypt

^10^Department of Agricultural Microbiology, Faculty of Agriculture, Ain Shams University, Cairo, Egypt

***Correspondence: Samy Selim (**[sabdulsalam@ju.edu.sa](mailto:sabdulsalam@ju.edu.sa)**)**

**Table S1:** Inhibition zone diameters (IZD, mm) of feather waste hydrolysate (FWH) and FWH-silver nanoparticles (FWH-AgNPs) against *Pseudomonas aeruginosa*, MRSA, *Aspergillus brasiliensis*, and *Candida albicans*.

| **Organism** | **FWH (mm) ± SD** | **FWH-AgNPs (mm) ± SD** | **Positive Control (mm) ± SD** | **Interpretation** |
| --- | --- | --- | --- | --- |
| ***P. aeruginosa*** | 36.0 ± 1.8ᵇ | 42.0 ± 2.1ᵃ | 46.5 ± 1.3 | Enhanced activity |
| **MRSA** | 31.0 ± 1.6ᵇ | 41.0 ± 2.0ᵃ | 44.0 ± 1.7 | Enhanced activity |
| ***A. brasiliensis*** | 19.0 ± 1.0ᵇ | 35.0 ± 1.8ᵃ | 38.5 ± 1.2 | Enhanced activity |
| ***C. albicans*** | 15.0 ± 0.8ᵇ | 28.0 ± 1.4ᵃ | 33.0 ± 1.6 | Enhanced activity |

*Values represent mean ± standard deviation from triplicate experiments (n = 3). Superscript letters (a, b) within each row indicate statistically significant differences (Student’s t-test, p < 0.05); different letters denote significant variation in antimicrobial activity, with ‘a’ indicating the higher effect.*

**Table S2.** Cytotoxic and viability responses of human skin fibroblasts (HSF) and MCF-7 breast cancer cells treated with FWH-AgNPs, assessed by MTT assay after 24 h exposure.

| **Concentration (µg/L)** | **HSF Viability (%) ± SD** | **MCF-7 Viability (%) ± SD** | **HSF Cytotoxicity (%) ± SD** | **MCF-7 Cytotoxicity (%) ± SD** |
| --- | --- | --- | --- | --- |
| **1000** | 76.8 ± 3.8ᵈ | 32.4 ± 2.1ᵃ | 23.2 ± 1.2ᵃ | 67.6 ± 3.5ᵉ |
| **500** | 83.5 ± 4.0ᶜ | 45.2 ± 2.4ᵇ | 16.5 ± 0.8ᵇ | 54.8 ± 2.7ᵈ |
| **250** | 87.4 ± 4.3ᵇ | 60.8 ± 2.9ᶜ | 12.6 ± 0.6ᶜ | 39.2 ± 2.1ᶜ |
| **125** | 90.2 ± 4.2ᵇ | 72.6 ± 3.5ᵈ | 9.8 ± 0.5ᶜ | 27.4 ± 1.4ᵇ |
| **62.5** | 93.6 ± 4.1ᵃ | 81.7 ± 3.8ᵉ | 6.4 ± 0.3ᵈ | 18.3 ± 0.9ᵃ |
| **31.25** | 95.2 ± 3.9ᵃ | 89.5 ± 4.1ᵉ | 4.8 ± 0.2ᵉ | 10.5 ± 0.6ᵃ |
| **IC₅₀ (µg/L)** | 790.3 | 294.7 | — | — |

**Data represent mean ± standard deviation (n = 3). Different superscript letters within each column indicate statistically significant differences at p < 0.05 (one-way ANOVA followed by Tukey’s post hoc test).**

**Table S3. Biochemical Disruptions Induced by Feather Waste Hydrolysate and Its Nanoparticle Formulation in *Culex pipiens* Larvae Over Time**

| **Time (h)** | **Treatment** | **Total Protein (µg)** | **Carbohydrate (µg)** | **AChE Activity (U)** |
| --- | --- | --- | --- | --- |
| **0** | Control | 12.00 ± 0.60ᵃ | 735.0 ± 36.75ᵃ | 5.28 ± 0.26ᶜ |
|  | FWH | 9.40 ± 0.47ᵇ | 620.0 ± 31.00ᵇ | 45.18 ± 2.26ᵃ |
|  | FWH-AgNPs | 5.20 ± 0.26ᶜ | 315.2 ± 15.76ᶜ | 25.78 ± 1.29ᵇ |
| **24** | Control | 7.50 ± 0.38ᵃ | 420.0 ± 21.00ᵃ | 45.26 ± 2.26ᵃ |
|  | FWH | 6.70 ± 0.34ᵇ | 350.0 ± 17.50ᵇ | 35.60 ± 1.78ᵇ |
|  | FWH-AgNPs | 3.10 ± 0.16ᶜ | 179.6 ± 8.98ᶜ | 19.50 ± 0.98ᶜ |
| **48** | Control | 3.24 ± 0.16ᵃ | 268.0 ± 13.40ᵃ | 35.12 ± 1.76ᵃ |
|  | FWH | 2.10 ± 0.11ᵇ | 220.0 ± 11.00ᵇ | 3.45 ± 0.17ᶜ |
|  | FWH-AgNPs | 1.80 ± 0.09ᶜ | 110.5 ± 5.53ᶜ | 18.50 ± 0.93ᵇ |
| **72** | Control | 0.195 ± 0.0098ᵃ | 115.0 ± 5.75ᵃ | 15.68 ± 0.78ᵇ |
|  | FWH | 0.120 ± 0.0060ᵇ | 95.15 ± 4.76ᵇ | 15.80 ± 0.79ᵇ |
|  | FWH-AgNPs | 0.090 ± 0.0045ᶜ | 50.25 ± 2.51ᶜ | 7.56 ± 0.38ᶜ |

(Data = mean ± SD per 25 larvae; different superscript letters indicate significant differences, *p* < 0.05, ANOVA with Tukey’s test)
